# Exploration of prognosis and immunometabolism landscapes in ER+ breast cancer based on a novel lipid metabolism-related signature

**DOI:** 10.3389/fimmu.2023.1199465

**Published:** 2023-07-04

**Authors:** Lesang Shen, Huanhuan Huang, Jiaxin Li, Wuzhen Chen, Yao Yao, Jianming Hu, Jun Zhou, Fengbo Huang, Chao Ni

**Affiliations:** ^1^ Department of Breast Surgery, Second Affiliated Hospital, Zhejiang University, Hangzhou, Zhejiang, China; ^2^ Key Laboratory of Tumor Microenvironment and Immune Therapy of Zhejiang Province, Second Affiliated Hospital, Zhejiang University, Hangzhou, Zhejiang, China; ^3^ Cancer Center, Zhejiang University, Hangzhou, China; ^4^ Department of Breast Surgery, Affiliated Hangzhou First People’s Hospital, Zhejiang University, Hangzhou, Zhejiang, China; ^5^ Department of Pathology, Second Affiliated Hospital, Zhejiang University, Hangzhou, Zhejiang, China

**Keywords:** lipid metabolism, estrogen receptor-positive breast cancer, prognostic signature, tumor immune microenvironment, therapy response

## Abstract

**Introduction:**

Lipid metabolic reprogramming is gaining attention as a hallmark of cancers. Recent mounting evidence indicates that the malignant behavior of breast cancer (BC) is closely related to lipid metabolism. Here, we focus on the estrogen receptor-positive (ER+) subtype, the most common subgroup of BC, to explore immunometabolism landscapes and prognostic significance according to lipid metabolism-related genes (LMRGs).

**Methods:**

Samples from The Cancer Genome Atlas (TCGA) database were used as training cohort, and samples from the Molecular Taxonomy of Breast Cancer International Consortium (METABRIC), Gene Expression Omnibus (GEO) datasets and our cohort were applied for external validation. The survival-related LMRG molecular pattern and signature were constructed by unsupervised consensus clustering and least absolute shrinkage and selection operator (LASSO) analysis. A lipid metabolism-related clinicopathologic nomogram was established. Gene enrichment and pathway analysis were performed to explore the underlying mechanism. Immune landscapes, immunotherapy and chemotherapy response were further explored. Moreover, the relationship between gene expression and clinicopathological features was assessed by immunohistochemistry.

**Results:**

Two LMRG molecular patterns were identified and associated with distinct prognoses and immune cell infiltration. Next, a prognostic signature based on nine survival-related LMRGs was established and validated. The signature was confirmed to be an independent prognostic factor and an optimal nomogram incorporating age and T stage (AUC of 5-year overall survival: 0.778). Pathway enrichment analysis revealed differences in immune activities, lipid biosynthesis and drug metabolism by comparing groups with low- and high-risk scores. Further exploration verified different immune microenvironment profiles, immune checkpoint expression, and sensitivity to immunotherapy and chemotherapy between the two groups. Finally, arachidonate 15-lipoxygenase (ALOX15) was selected as the most prominent differentially expressed gene between the two groups. Its expression was positively related to larger tumor size, more advanced tumor stage and vascular invasion in our cohort (n = 149).

**Discussion:**

This is the first lipid metabolism-based signature with value for prognosis prediction and immunotherapy or chemotherapy guidance for ER+ BC.

## 1 Introduction

Breast cancer (BC) is the most common tumor and predominant cause of tumor-related death among women worldwide (1). As the most common subtype, estrogen receptor-positive (ER+) BC comprises approximately 75% of BC cases (2). Although patients with ER+ BC have a relatively favorable prognosis compared to those with other subtypes, 30–40% are still at risk of relapse due to resistance to endocrine regimens or chemotherapy, which may be related to clinical and biological heterogeneity (3, 4). Thus, there is an urgent need to develop novel biomarkers to identify patients with high-risk ER+ BC and to optimize individual therapeutic strategies, which will ultimately lead to prolonged survival.

Lipids, comprising fatty acids, phospholipids, cholesterol, and triglycerides, are required for energy generation, membrane formation, and transduction of biological signals. In recent years, lipid metabolic reprogramming has progressively been recognized as a hallmark of malignancy (5, 6). Moreover, it has been recognized that lipid metabolism is intimately linked to oxidative stress, which often features a relative excess of reactive oxygen species (ROS) over antioxidants (7). For example, several lipid metabolism pathways are involved in ferroptosis, a unique iron-dependent cell death pathway characterized by oxidative stress and lipid peroxidation (8, 9). Previous metabolic studies have noted that each subtype of BC displays distinct metabolic alterations. Triple-negative BC (TNBC) tends to utilize exogenous fatty acids, whereas luminal subtypes appear to depend on a balance between oxidation and *de novo* fatty acid synthesis as energy sources, and human epidermal growth factor receptor 2-positive (HER2+) BC displays upregulated lipid biosynthesis (10–12). To date, there are no available studies focusing on the differences in lipid metabolism between specific BC subtypes.

The tumor microenvironment (TME) represents a unique metabolic niche that contains not only tumor cells but also stromal cells, immune cells and the extracellular matrix (13). Although ER+ BC is generally considered a “cold tumor” with low immune infiltration, a recent study using imaging mass cytometry revealed a subset of ER+ BCs containing immune-enriched areas (14). Therefore, it is meaningful to explore immune infiltration in ER+ BC, as immune infiltration patterns can be significantly associated with patient prognosis (15). Notably, an increasing number of studies have elucidated the relationship between dysfunctional immunity and abnormal lipid metabolism. For instance, lipid uptake mediated by the scavenger receptor CD36 was found to support regulatory T-cell (Treg) function and survival in the TME but impair the antitumor ability of CD8+ T cells through lipid peroxidation (16–18). Moreover, fatty acid oxidation (FAO) is required to activate immunosuppressive Treg cells and M2 macrophages and maintain memory T cells (19, 20). Nevertheless, a comprehensive depiction of the relationship between lipid metabolism and the TME in BC is currently lacking. In addition, lipid metabolism reprogramming of tumor cells markedly affects therapeutic efficiency (21). Because of the low sensitivity of ER+ BC to chemotherapy (22, 23), it is critical to identify a reliable biomarker to predict chemotherapy response and identify patients who are likely to benefit from neoadjuvant chemotherapy to avoid unnecessary treatment.

In the present study, a variety of bioinformatic approaches were used to examine the different features of lipid metabolism in ER+ BC. A reliable lipid metabolism-related gene (LMRG) signature for predicting the survival of patients with ER+ BC was established and validated. Then, we sought to explore the underlying relationships of this signature with the lipid metabolism landscape and TME factors, including immune infiltration and immune checkpoint expression. Moreover, the potential of our signature to predict immunotherapy and chemotherapy response was evaluated. Our results improve the understanding of the lipid metabolism features of ER+ tumors and promote individualized treatment for ER+ BC patients.

## 2 Materials and methods

### 2.1 Data acquisition

A total of 2780 ER+ BC samples from six independent datasets were included in this research. Transcriptome data and clinicopathologic information were obtained from The Cancer Genome Atlas (TCGA) database (802 ER+ BC cases), the Molecular Taxonomy of Breast Cancer International Consortium (METABRIC) database (1444 ER+ BC cases), and GSE7390 (134 ER+ BC cases), GSE1456 (62 ER+ BC cases), GSE25066 (298 ER+ BC cases), and GSE4779 (40 ER+ BC cases) from Gene Expression Omnibus (GEO). The specific clinical information can be found in **Table S1**. The raw microarray mRNA data were quantile-normalized and log2-transformed, and genes with more than one probe were averaged.

### 2.2 Collection of LMRGs

A total of 1034 LMRGs were extracted from one fatty acid metabolism hallmark gene set from the Gene Set Enrichment Analysis (GSEA) database, 12 LMRG sets from the Kyoto Encyclopedia of Genes and Genomes (KEGG) database, and 22 LMRG sets from the REACTOME database after removing overlapping genes (**Table S2**).

### 2.3 Unsupervised consensus clustering based on survival-associ1ated LMRGs

Univariate Cox regression analysis was first performed to identify overall survival (OS)-related LMRGs, and genes with a p value < 0.05 were selected for further evaluation. Unsupervised consensus clustering was applied to explore lipid metabolism-associated molecular characteristics of ER+ BC patients based on survival-related LMRGs using the “ConsensusClusterPlus” R package (24); 1000 iterations were performed to obtain stable classifications, and a maximum of k = 6 clusters were used.

### 2.4 Construction and validation of the LMRG-based prognostic signature

The log-rank test was used to identify LMRGs associated with unfavorable prognosis, and genes with a p value ≥ 0.05 were removed. Then, least absolute shrinkage and selection operator (LASSO) regression analysis was applied to select significant prognostic LMRGs and develop an LMRG-based risk signature. A risk score for each patient was calculated with a formula considering the optimized gene expression values (Ei) and estimated Cox regression correlation coefficients (βi) using the “glmnet” R package (25): risk score = ∑ Ei * βi. The survival risk score of our study was calculated as follows: Risk score = (0.2317 * HIBCH) + (0.2823 * OSBPL10) + (0.1183 * FIG4) + (0.2407 * OCRL) + (0.1499 * CPT1A) + (0.0423 * INPP5F) + (0.2273 * PTGES3) + (0.0177 * HSP90AA1) + (0.1636 * ALOX15). Univariate and multivariate Cox regression analyses, receiver operating characteristic (ROC) curve analysis and Kaplan‐Meier (K-M) analysis were used to check the stability and suitability of the model in the prediction of OS in four datasets separately.

### 2.5 DNA methylation of LMRGs

DNA methylation is a critical epigenetic modification affecting gene expression and cancer development (26). The correlation between DNA methylation and gene expression was analyzed by DNMIVE (27), and survival analysis based on single CpG methylation was performed with MethSurv (https://biit.cs.ut.ee/methsurv/) (28).

### 2.6 Development and evaluation of a lipid metabolism-related clinicopathologic nomogram

Independent prognostic predictors were identified using the Cox proportional hazards model. A novel lipid metabolism-related nomogram considering the risk score and two clinical factors (age and T stage) was constructed using TCGA cohort data with the “regplot” and “rms” R packages (29). Calibration curves of 1-, 3-, and 5-year OS were generated to assess the accuracy of our nomogram (30). To further examine the clinical significance of the risk score, boxplots of Wilcoxon test values were generated to visualize differences in risk scores across diverse clinicopathologic parameters.

### 2.7 Gene enrichment and pathway analysis

Gene Ontology (GO) biological process and KEGG pathway enrichment analyses of survival-related LMRGs and differentially expressed genes (DEGs) were performed with the “clusterProfiler” R package (31). GSEA was performed using GSEA software (version 4.2.3). To explore the correlation between the lipid metabolism-based signature and immune-related metagenes (32), gene set scores of samples for each cluster were calculated by gene set variation analysis (GSVA) in the “GSVA” R package (33). DEGs between the high- and low-risk groups with log2 (fold change) > 1 and p value < 0.05 were identified using the “limma” R package.

### 2.8 Estimation of the tumor immune microenvironment landscape

The CIBERSORT (34) and xCell (35) algorithms were used to quantify the relative abundances of tumor immune-infiltrating cells (TIICs) in tumor samples. Using the ESTIMATE algorithm (36), we also calculated the immune and stromal scores of ER+ BC patients from TCGA, which reflect enrichment of gene signatures related to immune and stromal cells. Moreover, according to a study by Thorsson et al. (37), we generated boxplots of risk scores in diverse immune and cancer subtypes.

### 2.9 Potential implications of the signature for immunotherapy

Great progress has been made recently in immunotherapy for BC, but reliable biomarkers for assessing response to immunotherapy still need to be identified. Therefore, we sought to predict the effect of immunotherapy based on the lipid metabolism-related signature. The expression levels of several immune checkpoints were assessed and compared between the two risk groups. These candidate checkpoints are listed in **Table S3**. Additionally, TCGA somatic mutation data for ER+ BC were downloaded. As a widely used predictor of the checkpoint inhibitor response (38), tumor mutation burden (TMB) was defined as the sum of all nonsynonymous mutations in each sample.

### 2.10 Prediction of chemotherapeutic response

The Genomics of Drug Sensitivity in Cancer (GDSC) database (39) and the Cancer Cell Line Encyclopedia (CCLE) database (40) were used to estimate individual chemotherapeutic responses. Three commonly used chemotherapy drugs for BC were selected: docetaxel, doxorubicin, and cisplatin. Then, the half-maximal inhibitory concentration (IC50) of each drug was assessed via the “pRRophetic” R package (41). Previous studies have proven that chemoresistance is associated with cancer stemness and chromosomal instability features, including copy number variation (CNV) (42–44). The mRNA stemness index (mRNAsi) was calculated as described by Malta et al. (45), and the most frequently mutated genes were identified using the “maftools” R package (46).

### 2.11 Connections between small molecules and DEGs

The Connectivity Map (CMap) database (https://clue.io/) (47), a compilation of reference gene expression profiles from human cells treated with small bioactive molecules or drug molecules, was used to discover possible connections between the DEGs and small compounds based on gene expression profile similarities.

### 2.12 Histological validation and clinical data collection

We collected formalin-fixed paraffin-embedded sections from 149 patients who underwent surgical treatment and were confirmed to have ER+ BC at the Second Affiliated Hospital of Zhejiang University School of Medicine from January 2014 to June 2017. The inclusion criteria were defined as follows: (1) BC as a primary cancer diagnosis; (2) histological confirmation of BC; (3) curative operation performed; (4) ER positivity determined via immunohistochemistry (IHC) staining; and (5) complete clinicopathologic information. A 2-mm tissue core containing the dominant tumor area was collected for tissue microarrays. Collection of samples and clinicopathological information was undertaken after receiving informed consent and approval by the ethics committee. Staining scores were calculated by multiplying the proportion of positively stained tumor cells by the staining intensity. The samples were classified as having no (0), < 25% (1), 25–50% (2), 50–75% (3), or 75–100% (4) positive cells. The intensity was classified as no staining (0), weak staining (1), moderate staining (2), or strong staining (3). IHC staining was performed on 4-μm-thick sections, as previously described. The anti-ALOX15 monoclonal antibody used was purchased from Abcam (ab244205). Images were photographed by laser confocal microscopy.

### 2.13 Statistical analysis

Statistical analyses were performed using GraphPad Prism (version 9; GraphPad Software) and R software (version 4.0.3); a two-tailed p value < 0.05 was considered statistically significant. The Wilcoxon rank-sum test was used to compare two groups. The K-M method and log-rank test were used to estimate prognosis. A Cox proportional hazards model was used for univariate and multivariate analyses. Correlation analysis was performed with the Pearson rank correlation test.

## 3 Results

### 3.1 Identification and clustering of survival-associated LMRGs

**Figure 1:**
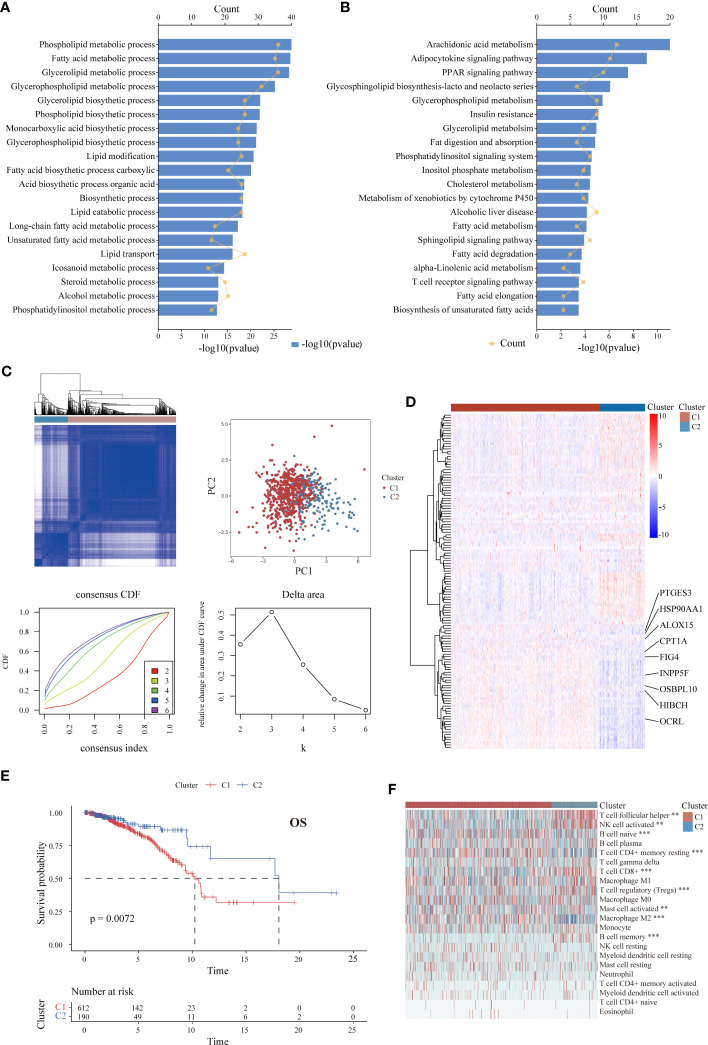
Identification of LMRG expression patterns associated with different prognoses and levels of immune infiltration in ER+ BC in the TCGA cohort. **(A, B)** GO and KEGG analyses of the 130 identified survival-associated LMRGs showing enrichment of lipid metabolic pathways. The top 20 pathways are presented. **(C)** Unsupervised consensus clustering showing two lipid metabolism-related clusters in ER+ BC. **(D)** Heatmap showing distinct expression patterns of these 130 LMRGs in two cluster subtypes. Some important genes are labeled. **(E)** K-M survival curve of patients stratified by cluster subtype. **(F)** Immune cell infiltration landscapes of the two cluster subtypes according to CIBERSORT. The abundances of some cell subpopulations significantly differed between the two clusters. **p < 0.01, ***p < 0.001.

The overall workflow of the present study is illustrated in **Figure S1**. A total of 802 patients with ER+ BC were included. Through univariate Cox analysis, 130 LMRGs were found to be significantly associated with OS (p < 0.05, **Table S4**). The top 20 most significant GO biological processes and KEGG pathways indicated that these genes are mainly involved in lipid metabolic pathways, including phospholipid and fatty acid metabolism (**Figures 1A, B**). Next, unsupervised consensus clustering was performed to explore the lipid metabolism-related patterns of ER+ BC according to the expression patterns of 130 survival-associated LMRGs. After comprehensive consideration of the unsupervised clustering patterns of the training and validation cohorts, the optimal number of clusters was two (k = 2) (**Figure 1C**). The patients were then divided into two subgroups: Cluster 1 (n = 612) and Cluster 2 (n = 190). The distinct expression patterns of the 130 significant LMRGs between the groups are represented as a heatmap in **Figure 1D**. Moreover, as demonstrated in K-M survival curves (**Figure 1E**), ER+ BC patients in Cluster 1 had significantly worse survival than those in Cluster 2 (p < 0.01). Although data for a few genes were absent in the validation cohorts, we still performed clustering analysis for each of the other cohorts using the same optimal k value. The OS results significantly differed between the groups (**Figure S2**). With the CIBERSORT algorithm, we systematically evaluated the abundance of 22 TIIC subpopulations in the samples from TCGA and found that the extent of immune cell infiltration varied between groups. For example, the levels of T follicular helper cells, CD8+ T cells and activated natural killer (NK) cells, which are correlated with a positive prognosis in BC (48), were higher in Cluster 2 (p < 0.01), whereas the levels of activated mast cells and M2 macrophages (49), which are correlated with a negative prognosis, were higher in Cluster 1 (p < 0.001) (**Figure 1F**).

### 3.2 Construction and validation of the LMRG-based prognostic signature for ER+ BC patients

**Figure 2:**
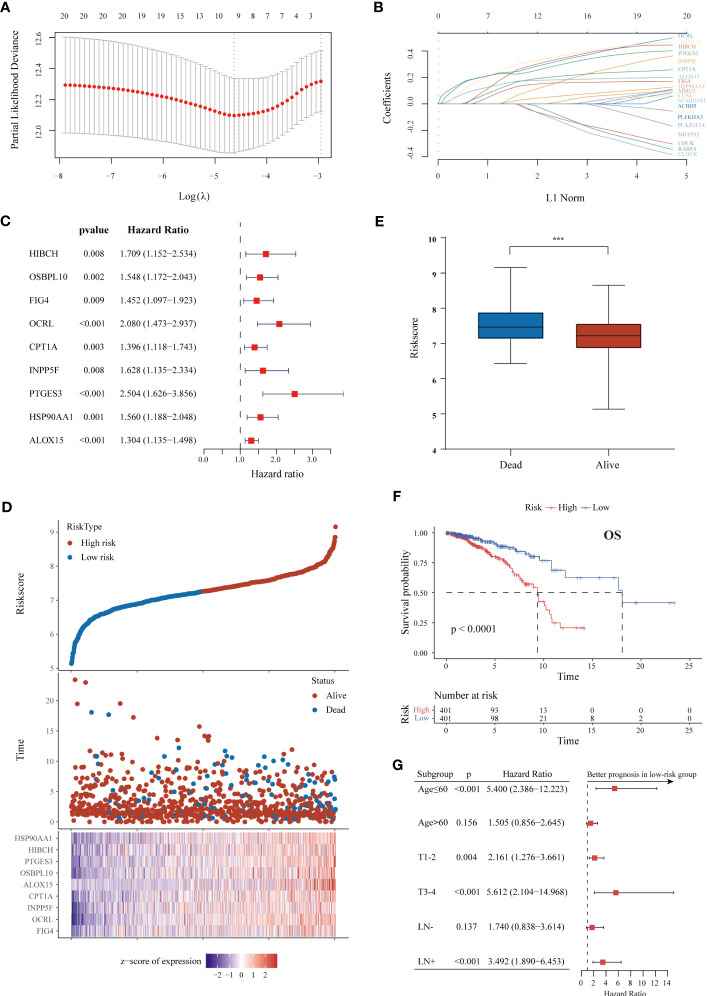
Construction of a survival-associated LMRG-based signature for ER+ BC. **(A, B)** LASSO coefficient profiles and cross-validation via minimum criteria to select significant prognostic LMRGs. **(C)** Forest plot of univariate Cox regression analysis results showing that the nine lipid metabolism genes used for signature construction were related to poor prognosis. **(D)** Distributions of risk scores, survival status and gene expression in individual patients from TCGA. As the risk score increases, the number of deaths and gene expression levels also increase. **(E)** The risk scores of patients who died were higher than those of patients who lived. **(F)** K-M curve of OS in ER+ BC patients from the TCGA cohort classified based on the risk score. **(G)** Forest plot showing survival differences between the high- and low-risk groups in subgroups stratified by age, tumor size and lymphatic metastasis. The superior prognosis of the low-risk group was maintained in all subgroups. ***p < 0.001.

To further identify critical LMRGs associated with tumor malignancy, we applied the log-rank test for the above candidate LMRGs, and 25 genes with log-rank p value < 0.05 and HR > 1 were selected after filtering (**Table S5**). Using LASSO analysis with TCGA data, we identified the nine most robust LMRGs (HIBCH, OSBPL10, FIG4, OCRL, CPT1A, INPP5F, PTGES3, HSP90AA1, and ALOX15) and used them to construct a prognostic signature (**Figures 2A–C**). DNA methylation status is closely correlated with gene expression and the prognosis of cancer patients (26). We thus evaluated the association between promoter methylation levels and mRNA levels of the above genes in BC. Negative correlations were found for OSBPL10, FIG4, INPP5F, PTGES3 and HSP90AA1, while a positive correlation was observed between ALOX15 promoter methylation and mRNA level (**Figure S3A**). Furthermore, the impact of single CpG methylation of these nine genes on BC patient prognosis was examined, and the results are listed in **Table S6**. We confirmed the significant association between the methylation β values of specific sites and the OS of BC patients (**Figure S3B**).

TCGA cohort patients were divided into high-risk and low-risk groups according to the survival risk score (shown in Methods) with the median score as the threshold. The different expression levels of the nine genes are detailed in **Figure 2D**. The proportion of patients who died was higher in the high-risk group than in the low-risk group (**Figure 2D**). Moreover, patients who died during the follow-up period had an increased risk score (**Figure 2E**). The prognostic value of the signature in ER+ BC was validated by performing K-M and time-dependent ROC analyses. The results showed that patients with low risk had a much better OS rate (p < 0.0001) (**Figure 2F**). The area under the curve (AUC) values for predicting 1-, 3-, 5-, and 10-year OS were 0.634, 0.663, 0.696, and 0.730, respectively. The LMRG-based signature had a better predictive value than any individual gene (**Figure S4A**).

We next performed stratification-based survival analysis of the model in various clinical subgroups stratified by age, tumor size and lymphatic metastasis. There were significant differences in OS between the two risk groups in nearly all subgroups (**Figure 2G**). The risk scores in different clinicopathological subgroups are also presented in **Figure S4B**. A higher risk score was correlated with more severe clinical parameters, including advanced N and AJCC stages.

To confirm the prognostic value of our LMRG-based signature developed based on the training set, three independent cohorts (METABRIC, GSE7390, and GSE1456) were used as validation cohorts. With the same risk score calculation formula and median risk score, patients with ER+ BC in the validation cohorts were segregated into low-risk and high-risk groups. In the survival analysis, patients in the low-risk group showed longer OS than those in the high-risk group in the METABRIC (p = 0.012), GSE7390 (p = 0.029), and GSE1456 (p = 0.0022) cohorts, which was consistent with the results for the training set (**Figure S5**).

### 3.3 Development and evaluation of a lipid metabolism-related clinicopathologic nomogram

**Figure 3:**
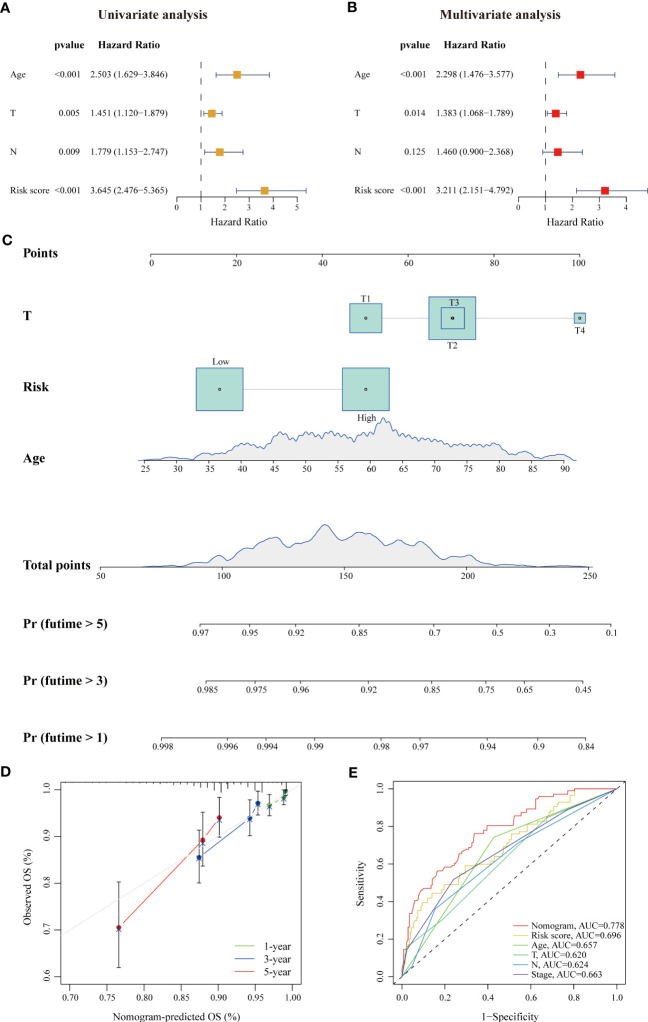
A risk-stratification-based clinicopathologic nomogram for OS prediction of patients with ER+ BC. **(A, B)** Univariate and multivariate Cox analyses of clinicopathologic factors and the risk score in ER+ BC patients in the TCGA cohort. Age, T stage and risk score were independent prognostic indicators. **(C)** Development of a prognostic nomogram considering the risk score, age and T stage to predict 1-, 3-, and 5-year OS in ER+ BC patients. **(D)** Calibration curve of the predicted and actual OS values, showing the stable performance of the nomogram. **(E)** ROC curves of clinicopathologic factors, the risk score, and the nomogram in predicting 5-year OS. The AUC values of each factor are shown.

For convenient clinical usage in early-stage BC patients, we simplified the risk score into a dichotomous variable (low/high). Then, we assessed this risk score and other clinicopathologic factors in univariate and multivariate Cox regression analyses using the training cohort (**Figures 3A, B**). Our results showed that age, pathological parameters (T and N) and the risk score were remarkably related to patient OS in the univariate Cox analysis (all p < 0.01); age (HR = 2.298, 95% CI: 1.476–3.577, p < 0.001), T stage (HR = 1.383, 95% CI: 1.068–1.789, p = 0.014) and the risk score (HR = 3.211, 95% CI: 2.151–4.792, p < 0.001) remained independent prognostic indicators of unfavorable OS. Based on these results, we developed a prognostic nomogram that incorporates the risk score and two other clinicopathologic factors (age and T stage) for predicting individual OS at 1, 3, and 5 years (**Figure 3C**), and calibration plots demonstrated the stable performance of the nomogram (**Figure 3D**). Moreover, our nomogram had better predictive accuracy than the AJCC staging system (AUC at 5 years: 0.778 versus 0.663) (**Figure 3E**). In summary, this nomogram based on the lipid metabolism-related risk score is useful for the survival prediction of ER+ BC patients.

### 3.4 Analysis of biological pathways and functions related to the LMRG-based signature

**Figure 4:**
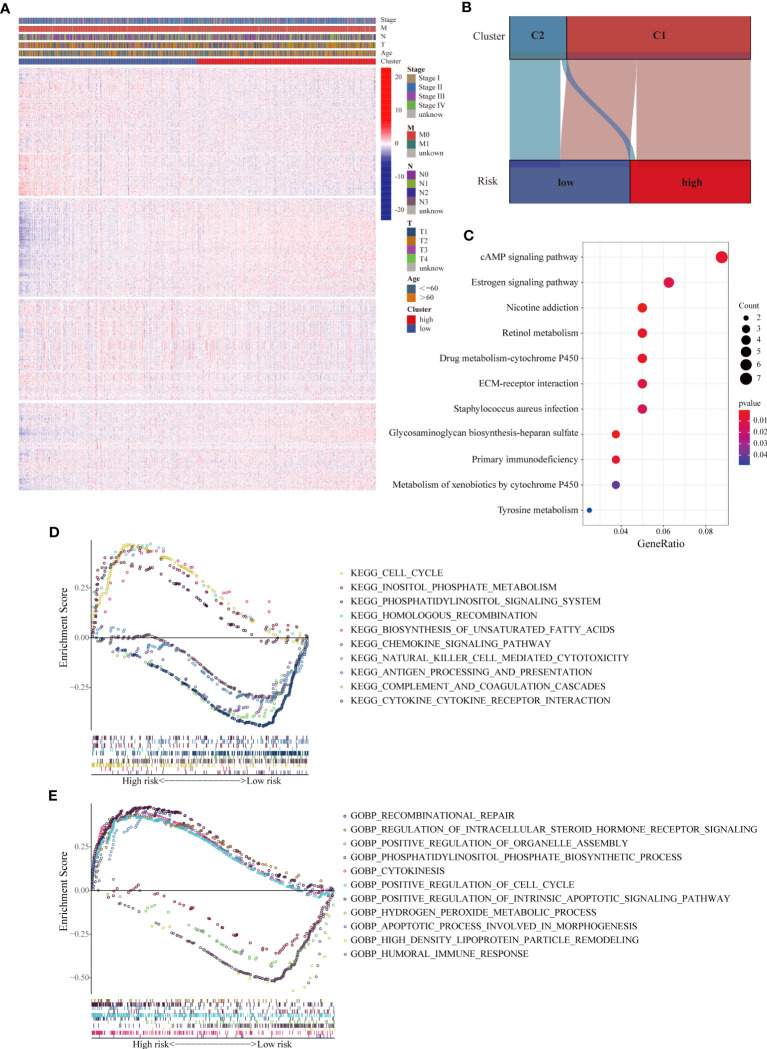
Analyses of biological processes and pathways related to the LMRG-based signature. **(A)** Heatmap of the expression levels of all 1034 LMRGs showing distinct expression patterns between the high- and low-risk groups from the TCGA cohort. **(B)** Correlations between the two clusters and the two risk groups. The majority of patients in Cluster 2 were categorized into the low-risk group. **(C)** KEGG pathway analysis of the DEGs between the two risk groups, revealing differentially activated pathways. **(D, E)** Representative KEGG pathways and GO biological processes enriched in the high- and low-risk patients, as determined by GSEA.

Given that the prognostic value of the LMRG-based signature was fully assessed, we attempted to explore the underlying mechanism. First, we evaluated the correlations between the expression levels of all the LMRGs and clinical parameters in various groups. Heatmap analysis showed remarkably distinct profiles of LMRG expression between the two groups (**Figure 4A**). In addition, most of the patients (88.9%) previously categorized in Cluster 2 were categorized into the low-risk group, and the majority of patients (62.1%) previously categorized in Cluster 1 were categorized into the high-risk group (**Figure 4B**). These results suggest that the signature based on the nine-gene risk score reflects the overall lipid metabolism characteristics of ER+ BC. We then performed differential expression analysis of the two risk groups and identified 133 upregulated genes and 92 downregulated genes in the high-risk group (**Table S7**). KEGG analysis revealed that the DEGs were enriched in several signaling pathways related to drug metabolism, immune factors, extracellular matrix interactions and estrogen (**Figure 4C**). We also performed GSEA to identify significantly differentially enriched biological functions and signaling pathways between the two groups from TCGA. Based on KEGG pathway and GO biological process analyses, the cell cycle, phospholipid metabolism, and unsaturated fatty acid biosynthesis pathways were enriched in high-risk patients, and antigen processing and presentation, immune response, and chemokine signaling pathways were enriched in low-risk patients (**Figures 4D, E**). These results suggest that the relationship of our established signature with immune activities, lipid biosynthesis and drug metabolism enable it to predict the survival of ER+ BC patients.

### 3.5 Immune microenvironment landscapes and immunotherapy response prediction

**Figure 5:**
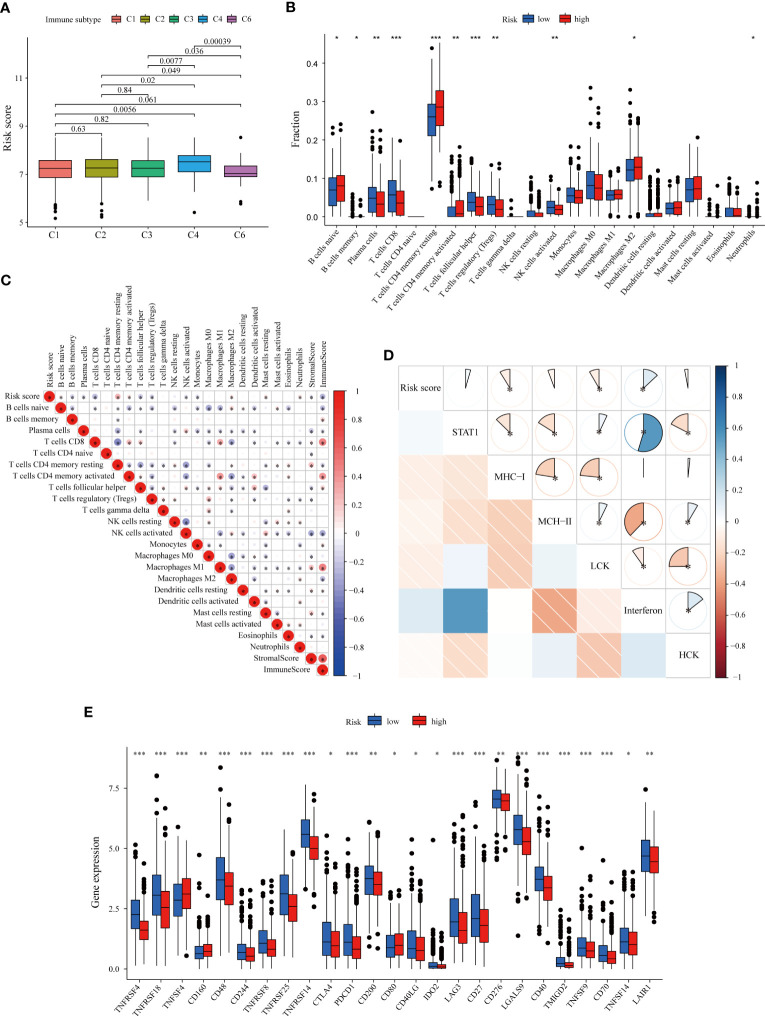
Immune microenvironment patterns and immune checkpoint profiles related to the LMRG-based signature in ER+ BC patients in the TCGA cohort. **(A)** Correlation between the risk score and pantumor immune subtypes. The C4 subtype (lymphocyte depletion) displayed the highest risk score. **(B)** Comparison of immune cell infiltration levels calculated according to CIBERSORT analysis between the two risk groups. **(C)** Correlation heatmap showing the correlations between immune cell infiltration levels and the LMRG-based risk score. The risk score was negatively correlated with the immune score estimated by the ESTIMATE algorithm. **(D)** Correlogram showing the correlations between the risk score and the six metagenes (STAT1, MHC-I, MHC-II, LCK, interferon, and HCK), which reflect inflammatory responses. **(E)** The expression levels of most immune checkpoints were higher in the low-risk group. *p < 0.05, **p < 0.01, ***p < 0.001.

Considering the close correlation between the LMRG-based signature and the immune response, we further explored the difference in the risk score of samples between different immune subtypes based on pantumor immunogenomic features (37). In particular, the risk score for the C4 subtype (lymphocyte depletion) was significantly higher than that for the other subtypes (**Figure 5A**), and the C4 subtype has been reported to be associated with a prominent macrophage signature and worse prognosis (37). We next evaluated the infiltration levels of diverse TIICs between the two risk groups to reveal differences in the immune microenvironment. According to our results, infiltrating naïve B cells, resting and activated memory CD4+ T cells, and M2 macrophages were more abundant in tumors of the high-risk group (p < 0.05). Furthermore, immune cells with antitumor function, including plasma cells, CD8+ T cells, follicular helper T cells and activated NK cells, were more abundant in the low-risk group (p < 0.05) (**Figure 5B**). Analysis was then performed to assess the correlation between the abundance of TIICs and the LMRG-based risk score. The risk score was strongly negatively correlated with the immune score calculated with the ESTIMATE algorithm, indicating greater immune infiltration in tumors with lower risk scores (**Figure 5C**). The immune infiltration profiles of the high- and low-risk groups were similar in the METABRIC cohort (**Figure S6A**). Inflammatory responses are tightly associated with immune functions (50). To further reveal risk score-related inflammatory activities, 91 genes derived from six clusters were defined as metagenes (STAT1, MHC-I, MHC-II, LCK, interferon, and HCK) (**Table S8**) (32, 51, 52). Using the “GSVA” R package, the scores of each sample based on the six metagene sets were calculated, with a higher score indicating a higher degree of enrichment. Correlation analysis revealed that the risk score was negatively correlated with MHC-I and LCK but positively correlated with interferon (**Figure 5D**), confirming that the low-risk group had higher antigen presentation and T-cell signatures.

Next, we evaluated the relationship between individual genes and the infiltration of various immune cell subpopulations. Through CIBERSORT analysis, we found that most selected genes were negatively correlated with the abundances of follicular helper T cells, CD8+ T cells, plasma cells, activated NK cells, memory B cells and Treg cells but positively correlated with the abundances of resting and activated memory CD4+ T cells, neutrophils, eosinophils and M2 macrophages (**Figure S6B**), consistent with the previously identified trends (**Figure 5C**). We also used the xCell algorithm as an alternative method to assess immune infiltration (**Figure S7**). Similarly, most of the nine genes were found to have negative correlations with immune cell infiltration; the expression levels of OCRL and PTGES3 were also negatively correlated with the immune score, indicating the close relationship between the LMRGs and “cold tumors”.

Subsequently, we investigated the correlation of the nine gene-based signature with immune checkpoints and its potential role in predicting response to immunotherapy. The expression levels of several immune checkpoints were compared between the two groups in the training set, and the molecules that were significantly differentially expressed between the groups are shown in **Figure 5E**. Most of the analyzed immune checkpoint genes, including CTLA-4, PDCD1 (PD-1), LAG3, IDO2 and CD276, were highly expressed in the low-risk group (p < 0.05). Similar patterns were also found for the METABRIC cohort (**Figures S6C, D**). TMB is an emerging biomarker for predicting response to immunotherapy (38). However, no significant differences in TMB were found (**Figure S8A**). Collectively, these results comprehensively reveal distinct immune features between high- and low-risk BC, with patients bearing low-risk tumors being more likely to benefit from immune checkpoint inhibitors.

### 3.6 Chemotherapy response prediction

**Figure 6:**
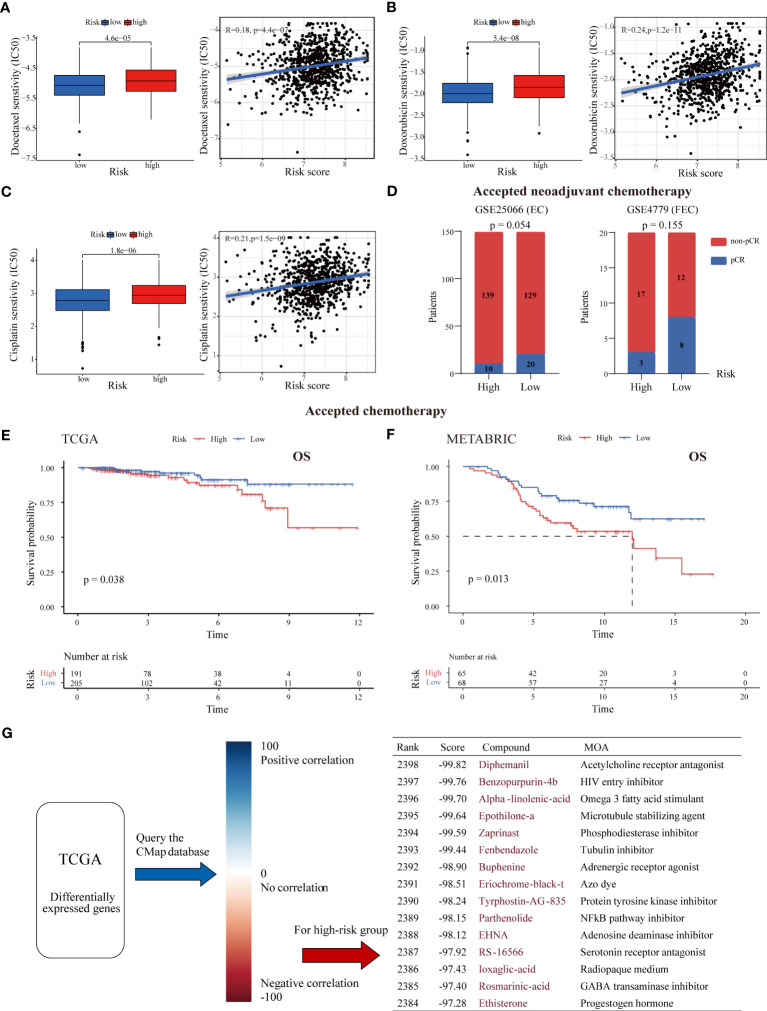
Association between the risk score and chemotherapy response and drug screening for high-risk tumors. The estimated IC50 values of docetaxel **(A)**, doxorubicin **(B)**, and cisplatin **(C)** in the low- and high-risk groups. High-risk tumors were more likely to be resistant to chemotherapy. **(D)** The pCR rate after receipt of neoadjuvant chemotherapy in the two risk groups from the GSE25066 and GSE4779 cohorts. ER+ BC patients with low risk were more likely to achieve pCR. **(E, F)** K-M survival curves of ER+ BC patients who received chemotherapy stratified by risk score in the TCGA and METABRIC cohorts. **(G)** CMap analysis of high-risk versus low-risk patients. The DEGs between the two risk groups were uploaded into the CMap database to predict potential drug targets. The top 15 drugs (with negative correlations) for treating high-risk tumors are listed.

Due to the enrichment of drug metabolism identified in the high-risk group and the importance of chemotherapy in BC treatment, we further investigated the association between our LMRG-based signature and chemotherapy efficacy (41). Three chemotherapeutic regimens (docetaxel, doxorubicin and cisplatin) commonly used in clinical practice were included in the assessment. Our results showed that the estimated IC50 values of all three drugs were significantly higher in high-risk tumors than in low-risk tumors (p < 0.001), suggesting that ER+ BC patients with higher risk scores are more resistant to cytotoxic chemotherapy (**Figures 6A–C**). To further validate these drug sensitivity results in BC cell lines, we assessed the correlations between the expression levels of each gene in each BC cell line and the IC50 values of chemotherapeutic drugs using the CCLE database. The results showed that most genes were associated with cell line resistance to docetaxel, doxorubicin and cisplatin, though there were no data for two genes (HIBCH and INPP5F) in the CCLE database (**Figure S8B**). Furthermore, as demonstrated in two independent cohorts, ER+ BC patients in the high-risk group had a much lower pathological complete response (pCR) rate than those in the low-risk group after receiving neoadjuvant chemotherapy (6.7% versus 13.4% in GSE25066; 15.0% versus 40.0% in GSE4779) (**Figure 6D**). ER+ BC patients who received chemotherapy in the high-risk group were found to have a worse prognosis in both the TCGA and METABRIC cohorts (p < 0.05) (**Figures 6E, F**). Notably, the LMRG-based risk score was positively associated with the stemness index, with a higher stemness index indicating a more aggressive phenotype (**Figure S8C**), whereas somatic mutation analysis showed no detectable differences in mutation rate between the two groups (83.56% and 85.29%) (**Figures S8D, E**). These results indicate that our signature might be able to predict chemotherapy response and the efficacy of neoadjuvant chemotherapy.

Next, the DEGs between the high- and low-risk groups were subjected to CMap analysis to identify candidate small molecule compounds for the treatment of high-risk tumors (**Figure 6G**). The top 15 drugs with high negative correlations in the ER+ BC cell line MCF7 were obtained, including the microtubule stabilizing agent epothilone A, the phosphodiesterase inhibitor zaprinast, the NF-kB pathway inhibitor parthenolide and the adenosine deaminase inhibitor EHNA. Notably, some cyclin-dependent kinase (CDK) inhibitors and estrogen receptor agonists were also near the top of the list. These results may provide a reference for choosing antitumor therapies for high-risk BC patients.

### 3.7 IHC analysis of ALOX15 expression in ER+ BC primary tumors

To further explore the value of individual DEGs between the high- and low-risk groups, upregulated genes in the high-risk group were ranked according to fold change and false discovery rate (FDR) (**Figure S9A**). We present the top 10 upregulated genes ordered by fold change and FDR in **Table S7**. By taking the intersection of these two gene lists, ALOX15, which was also included in our lipid metabolism-related signature, was obtained. As expected, high ALOX15 expression was associated with poor OS in ER+ BC patients (**Figure S9B**). As there is a paucity of research on the biological features of ALOX15 in BC, we further assessed ALOX15 expression in ER+ BC through histological analysis in our cohort.

To determine the expression of ALOX15, IHC analysis was performed on tissue microarray slides comprising 149 individual tumor tissues from ER+ BC patients with clinicopathological information. ALOX15 expression was heterogeneous and mainly located in the cytoplasm of cancer cells (**Figure S9C**). The patients were classified into high- and low-expression groups based on the median ALOX15 expression level. The results showed that high expression of ALOX15 was significantly positively associated with larger tumor size (p = 0.013) and vascular invasion (p = 0.043) and correlated with advanced tumor stage with borderline significance (p = 0.060) (**Table 1**), suggesting that ALOX15 is related to the malignant behavior of BC.

**Table 1:** Clinical and pathological characteristics of the ALOX15^high^ and ALOX15^low^ ER+ groups in our cohort.

Variables	ALOX15^high^	ALOX15^low^	p
Number of patients	70 (47.0%)	79 (53.0%)	
Age at diagnosis (years)	55.28 ± 10.82	55.33 ± 10.72	0.964
HER2 status			0.989
Positive	15 (21.4%)	17 (21.5%)	
Negative	55 (78.6%)	62 (78.5%)	
T stage			**0.013**
I	38 (54.3%)	58 (73.4%)	
II	28 (40.0%)	21 (26.6%)	
III	4 (5.7%)	0 (0.0%)	
N stage			0.219
N0	42 (60.0%)	55 (69.6%)	
N1–N3	28 (40.0%)	24 (30.4%)	
Stage			0.060
I	28 (40.0%)	47 (59.5%)	
II	29 (41.4%)	22 (27.8%)	
III	13 (18.6%)	10 (12.7%)	
Ki-67	0.20	0.19	0.653
Histological grade*			0.382
G1	3 (4.8%)	4 (5.8%)	
G2	52 (82.5%)	61 (88.4%)	
G3	8 (12.7%)	4 (5.8%)	
Vascular invasion*			**0.043**
Yes	23 (36.5%)	15 (20.8%)	
No	40 (63.5%)	57 (79.2%)	

* Some patients were not evaluated. The bold values mean that they are less than 0.05.

In addition, we evaluated the prognostic value of ALOX15 in our cohort. During a median follow-up time of 64 months, only 14 tumor relapse events and 2 death events occurred, and this was mainly due to the early stage of the patients (84.6% with stage I/II BC and 65.1% with negative lymph nodes, **Table 1**). However, 71.4% (10/14) of recurrence events occurred in patients with high ALOX15 expression in our cohort, and we validated the close association between ALOX15 expression and unfavorable pathological parameters (higher T stage and tumor stage) in the METABRIC dataset (**Figure S9D**).

## 4 Discussion

The role of oxidative stress in tumorigenesis, metastasis and cancer immunity has been well documented (7). In recent years, lipid metabolic reprogramming, which plays an essential role in tumor growth and progression, drug resistance, TME reprogramming and immune dysregulation, has gained increasing attention in the field of cancer research (6, 53). Because it leads to the production of ROS and mediates ferroptosis, lipid metabolism is critical for the regulation of oxidative stress (54). Although evidence indicates that there is aberrant lipid metabolism in BC (11), studies exploring the metabolic heterogeneity among specific BC subtypes are still lacking. Here, we established a lipid metabolism-related signature for ER+ BC with potential value in predicting survival, immune infiltration and therapy response.

First, we constructed an optimal and robust nine-LMRG prognostic signature. Most of the included genes have been reported to exert protumor functions and are associated with prognosis in a variety of malignant diseases, including BC (55–59). For example, CPT1A, the rate-limiting enzyme during FAO, has been found to promote cell proliferation and survival in luminal BC (60, 61). Serum CPT1A levels are also associated with the tumor burden of BC (62). HSP90AA1, one of the multifunctional HSP90 isoforms that plays a role in folding or stabilizing proteins such as cell cycle regulatory proteins and steroid hormone receptors, is associated with the prognosis of patients with ER+ BC and immune infiltration in the BC microenvironment (63, 64). In addition, Wang et al. suggested that BC cells express PTGES to generate a local immunosuppressive environment through myeloid-derived suppressor cell (MDSC) recruitment, which impairs the cytotoxic function of CD8+ T cells (65). Some of the included genes (such as FIG4, OCRL and INPP5F) belong to the group of phosphoinositide phosphatases, the members of which participate in a variety of cellular biological processes as signaling molecules (66). Among them, INPP5F has been recently demonstrated to be an oncogene that activates the ASPH-mediated Notch-c-MYC/cyclin E1 pathway in hepatocellular carcinoma (57). As a crucial enzyme of valine catabolism, HIBCH was reported to promote oxidative phosphorylation and tumor growth in colorectal cancer (55). ALOX15 expression in BC metastatic lymph nodes has been found to inversely correlate with metastasis-free survival (67), but its biological and clinical significance in primary lesions of BC is still unknown. In the present study, the expression of ALOX15 in primary lesions was found to be associated with malignant pathological features of ER+ BC. In addition, considering the limited utility for prognosis prediction that can be derived from the expression of a single gene (**Figure S4A**), we divided patients into high- and low-risk groups based on the median risk score and treated the risk score as a dichotomous variable for nomogram development. We believe this predictive model has greatly superior applicability across patients and practicability and will help identify high-risk patients with ER-positive expression.

The TME is strongly influenced by local lipid metabolism (68). Here, we revealed that tumor immunity differed between the high- and low-risk groups defined based on the LMRG signature. Although ER+ BC is generally considered a “cold tumor”, recent genomic, proteomic and single-cell studies have revealed heterogeneity and the existence of an activated immune phenotype in luminal BC with enrichment of TIICs (14, 52, 69). In our findings, low-risk tumors contained significant infiltration of immune cells, including CD8+ T cells and activated NK cells, as well as high MHC-I expression. However, high-risk tumors showed an “immune-desert” phenotype, including low lymphocyte infiltration and a high abundance of M2 macrophages. Accumulated evidence suggests that several immune cell subpopulations exhibit an immunosuppressive phenotype to shift metabolic patterns toward lipid metabolism, such as FAO (70, 71). Multiple lipid metabolism modulators have antitumor and immunomodulatory capacities (72). For instance, inhibition of FAO by etomoxir, a specific inhibitor of CPT1A, blocks the immune-suppressive abilities of tumor-infiltrating MDSCs, resulting in T-cell-dependent tumor growth restriction (73). In melanoma, inhibition of lipid synthesis and metabolic signaling by targeting SREBPs in Tregs could effectively activate antitumor immune responses without causing autoimmune toxicity (74). Therefore, our results provide potential targets to reverse aberrant lipid metabolism and improve therapeutic efficacy.

Finally, abnormal lipid metabolism is associated with reduced oxidative and endoplasmic reticulum stress in tumor cells and counteraction of genotoxicity or maintenance of drug-resistant stem cells (75). For instance, STAT3, a key regulator of lipid metabolism, promotes BC cell stemness and chemoresistance via the STAT3-CPT1B-FAO pathway (76). These results are consistent with our results, with high-risk patients predicted to be more resistant to docetaxel, doxorubicin and cisplatin. In addition, residual cancer cells surviving after neoadjuvant treatment have been observed to have elevated and dysregulated lipid metabolism (77), in agreement with our finding that the low-risk group had a much higher pCR rate. Recently, the ISPY-2 trial revealed that the combination of chemotherapy and pembrolizumab yielded a twofold increase in the pCR rate compared with chemotherapy alone in the ER+ subgroup (28% versus 14%) (78), indicating that the combination of chemotherapy and immune checkpoint inhibitors produces a synergistic antitumor effect in ER+ BC. Based on our study, patients in the low-risk group may benefit more from chemotherapy, immunotherapy, or their combination, indicating the predictive value of our risk model in the neoadjuvant treatment setting. Moreover, as high-risk tumors had enrichment of pathways related to fatty acid biosynthesis, the cell cycle and estrogen signaling, a synergistic effect might be anticipated by combining regimens targeting lipid metabolism with CDK inhibitor-based endocrine therapy.

Overall, we employed diverse bioinformatic approaches to unveil the heterogeneity of lipid metabolism in ER+ BC, primarily relying on transcriptome data. There are already numerous standardized pipelines available as references for RNA-seq data analysis (79, 80). Except for “Consensus Clustering” we utilized, “non-negative matrix factorization (NMF)” is also a robust approach for uncovering transcriptional clustering (81, 82). Besides evaluating tumor-infiltrating immune cells through single-cell sequencing, several quantitative analysis methods based on bulk sequencing data have been extensively applied. These methods primarily involve the utilization of single-sample GSEA with marker genes, such as xCell (35) and ImmuCellAI (83), as well as deconvolution-based methods like CIBERSORT (34), TIMER2.0 (84) and EPIC (85).

Several limitations of this study need to be acknowledged. First, although we used many training and validation datasets to verify the predictive potential of our signature, all included cohorts were retrospective, and the findings should be validated by biomarker analysis in prospective studies with large sample sizes. Second, signature genes were selected based on bioinformatic approaches, and understanding the underlying mechanism, especially how these genes influence the lipid metabolism of cancer cells or immune and stromal cells, requires future *in vitro* and *in vivo* studies. Third, our follow-up (median 64 months) was not long enough to collect enough recurrence or death events for prognostic analysis.

In conclusion, we constructed a survival-associated LMRG-based signature for ER+ BC for the first time, and we revealed underlying relationships between our signature and tumor immunity and therapeutic sensitivity. Future prospective clinical trials with large sample sizes are required to confirm the application value of the LMRG-based signature.

## Data availability statement

The original contributions presented in the study are included in the article/**Supplementary Material**. Further inquiries can be directed to the corresponding authors.

## Ethics statement

The studies involving human participants were reviewed and approved by the Second Affiliated Hospital of Zhejiang University School of Medicine. Written informed consent for participation was not required for this study in accordance with the national legislation and the institutional requirements

## Author contributions

LS and CN designed the study. LS, WC and FH obtained and assembled the data. LS and HH drafted the manuscript. JL, YY, JH, JZ and CN revised the manuscript. All authors contributed to the article and approved the submitted version.
