# Supplementary material for: Exploration of prognosis and immunometabolism landscapes in ER+ breast cancer based on a novel lipid metabolism-related signature
Source: Front Immunol. 2023 Jul 4;14:1199465. doi: 10.3389/fimmu.2023.1199465 (PMC10352658; doi:10.3389/fimmu.2023.1199465)
Supplement: Supplementary file 1 [file DataSheet_1.docx]

Supplementary Material

Exploration of Prognosis and Immunometabolism Landscapes in ER+ Breast Cancer Based on a Novel Lipid Metabolism-Related Signature

Lesang Shen^1,2,3†^, Huanhuan Huang^1,2,3†^, Jiaxin Li^1,2,3†^, Wuzhen Chen^1,2,3^, Yao Yao^1,2,3^, Jianming Hu^1,2,3^, Jun Zhou^4^, Fengbo Huang^5^*, Chao Ni^1,2,3^*

*** Correspondence:** Chao Ni: drnichao@zju.edu.cn; Fengbo Huang: 2515183@zju.edu.cn

# Supplementary Figures and Tables

## Supplementary Figures


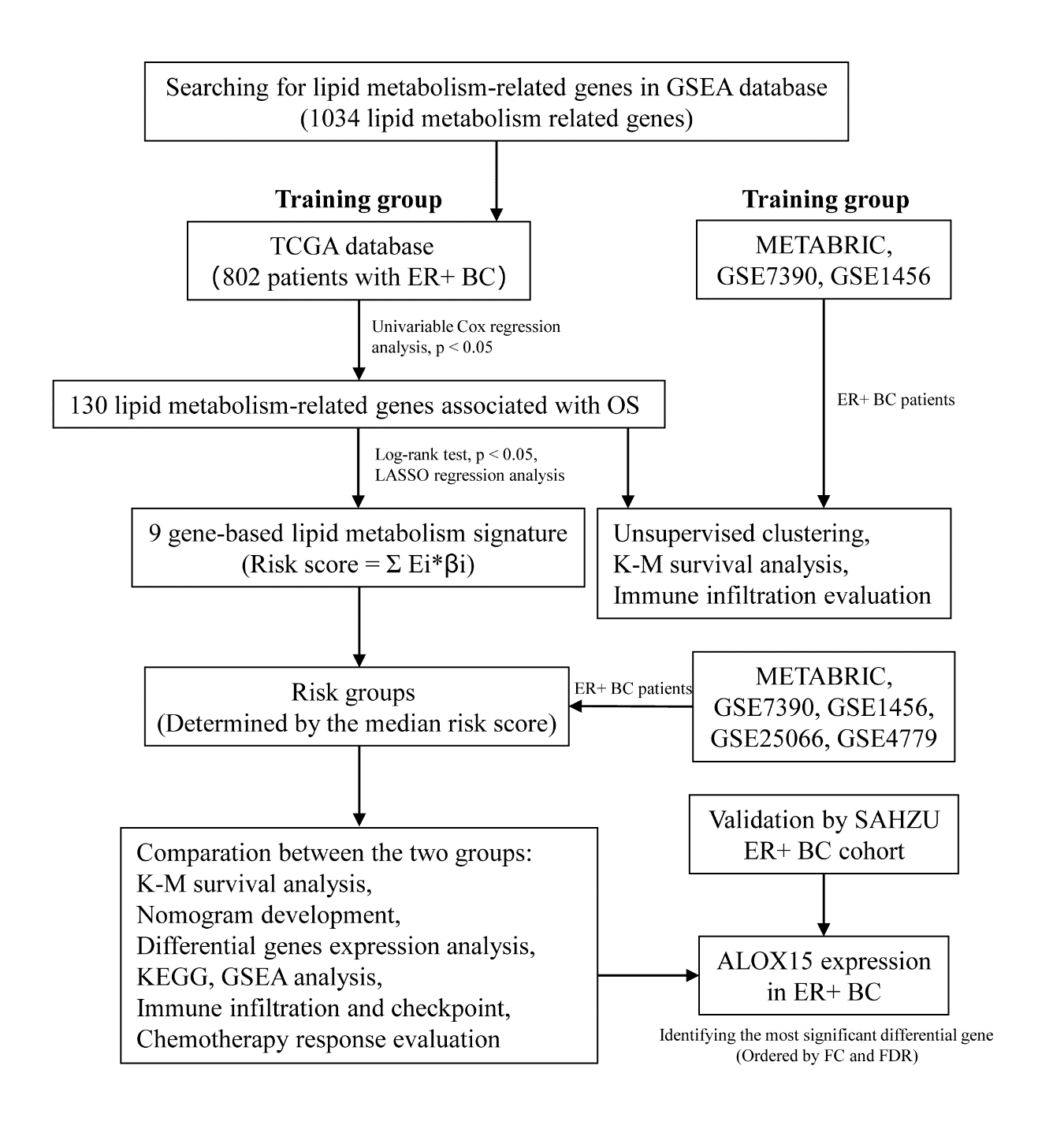


**Supplementary Figure 1.** Workflow of the study.

**
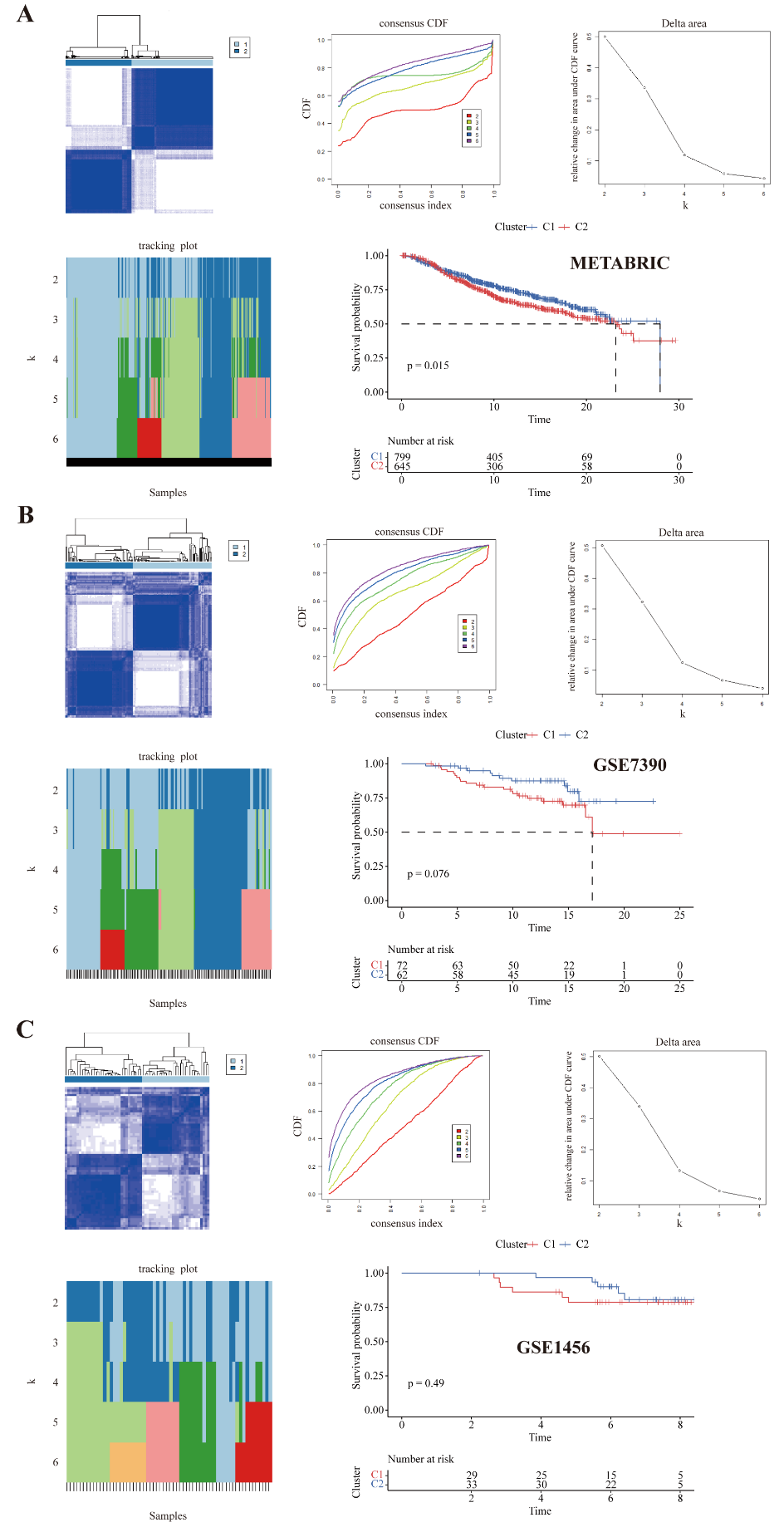
**

**Supplementary Figure 2.** Consensus clustering findings by the optimal cluster number and K-M curve survival analysis of patients stratified by cluster subtype in different validation cohorts, including METABRIC (A), GSE7390 (B), and GSE1456 (C).

**
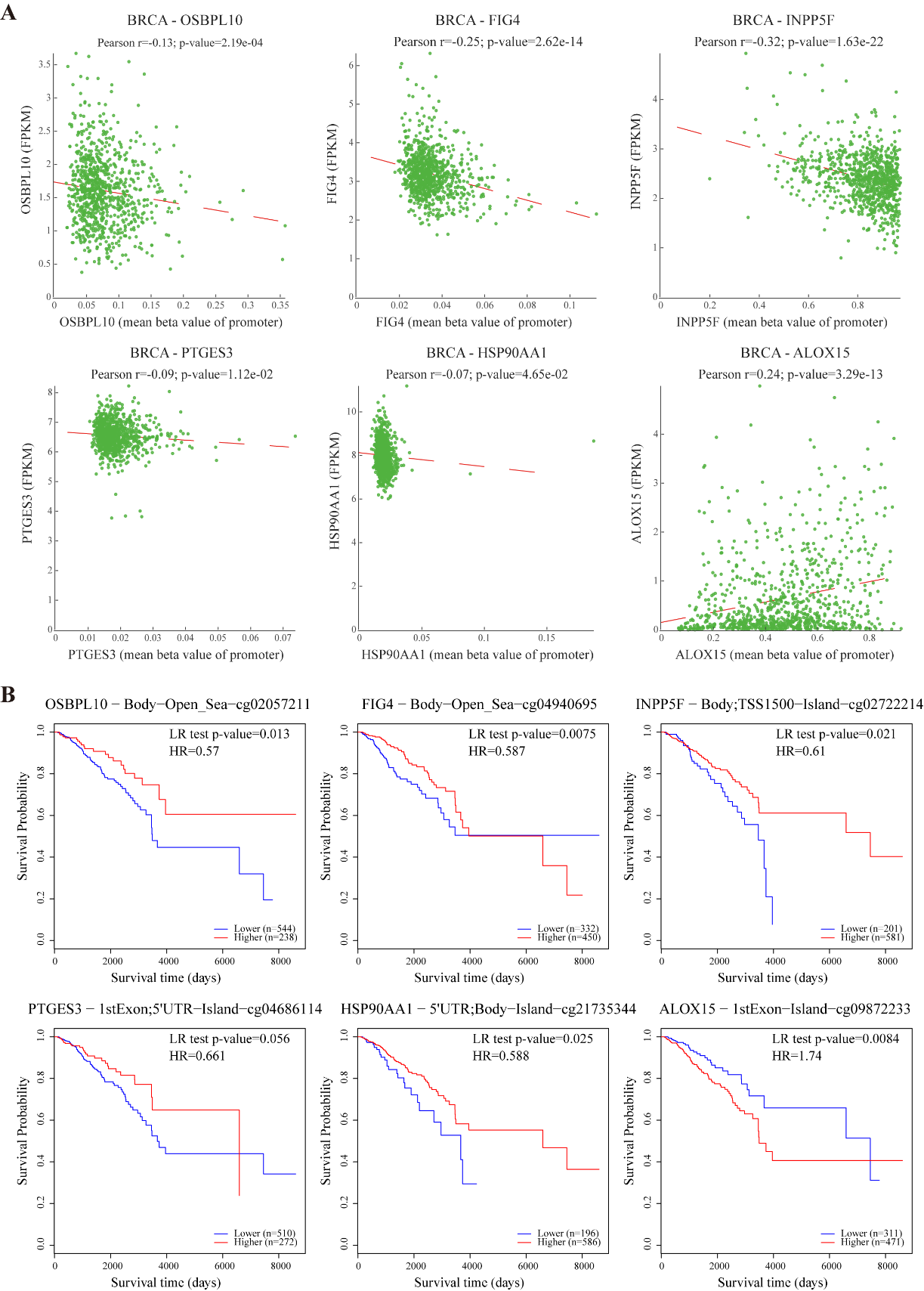
**

**Supplementary Figure 3.** DNA methylation of LMRGs influences gene expression and prognosis in BC. (A) Correlation between the promoter methylation and gene expression. (B) The impact of single CpG methylation on the OS of BC patients.

**
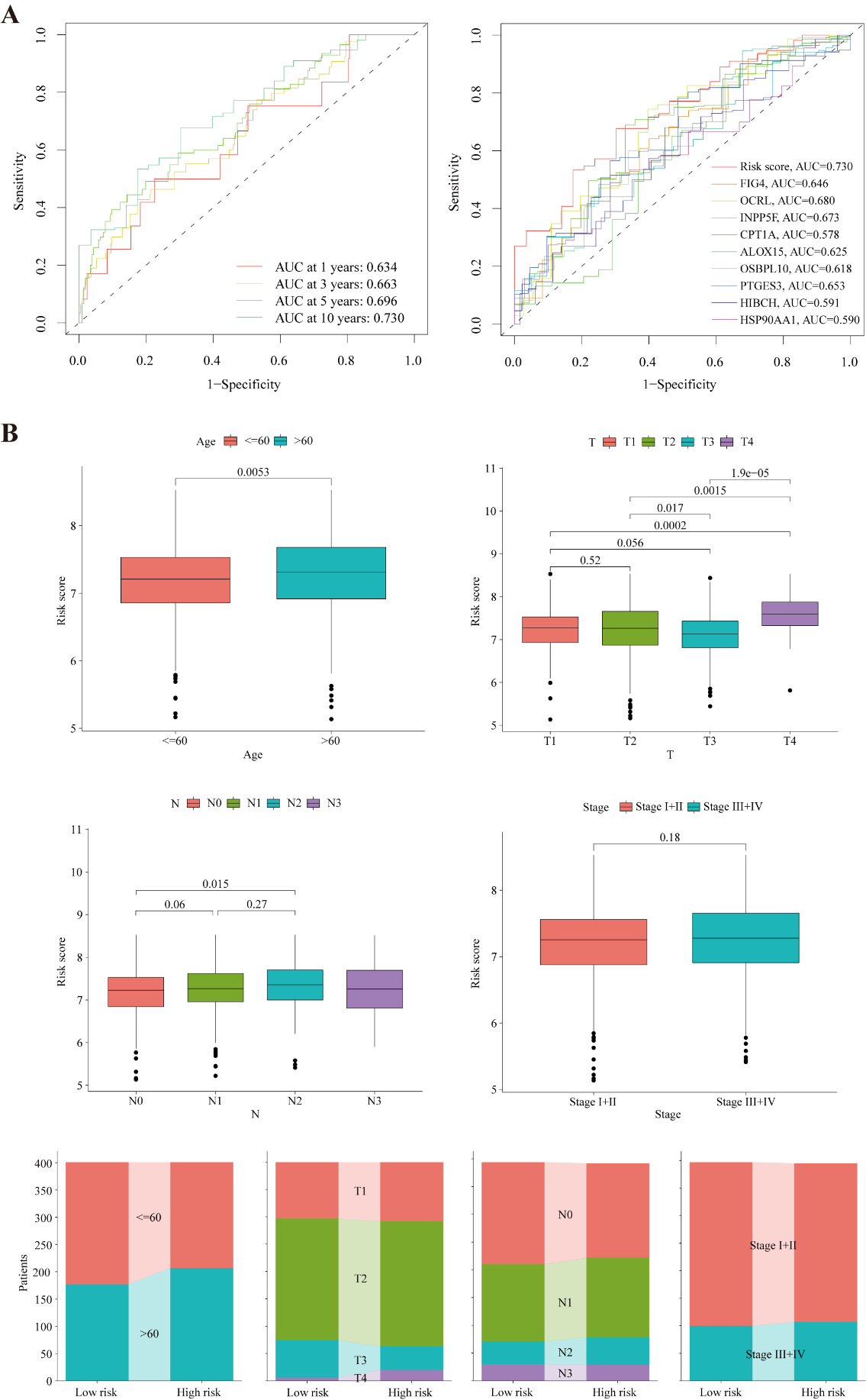
**

**Supplementary Figure 4.** (A) ROC curves of the risk score in predicting 1-, 3-, 5-, and 10-year OS (left). ROC curves of individual LMRGs in predicting 10-year OS (right). (B) Levels of risk score in different clinicopathological subgroups.

**
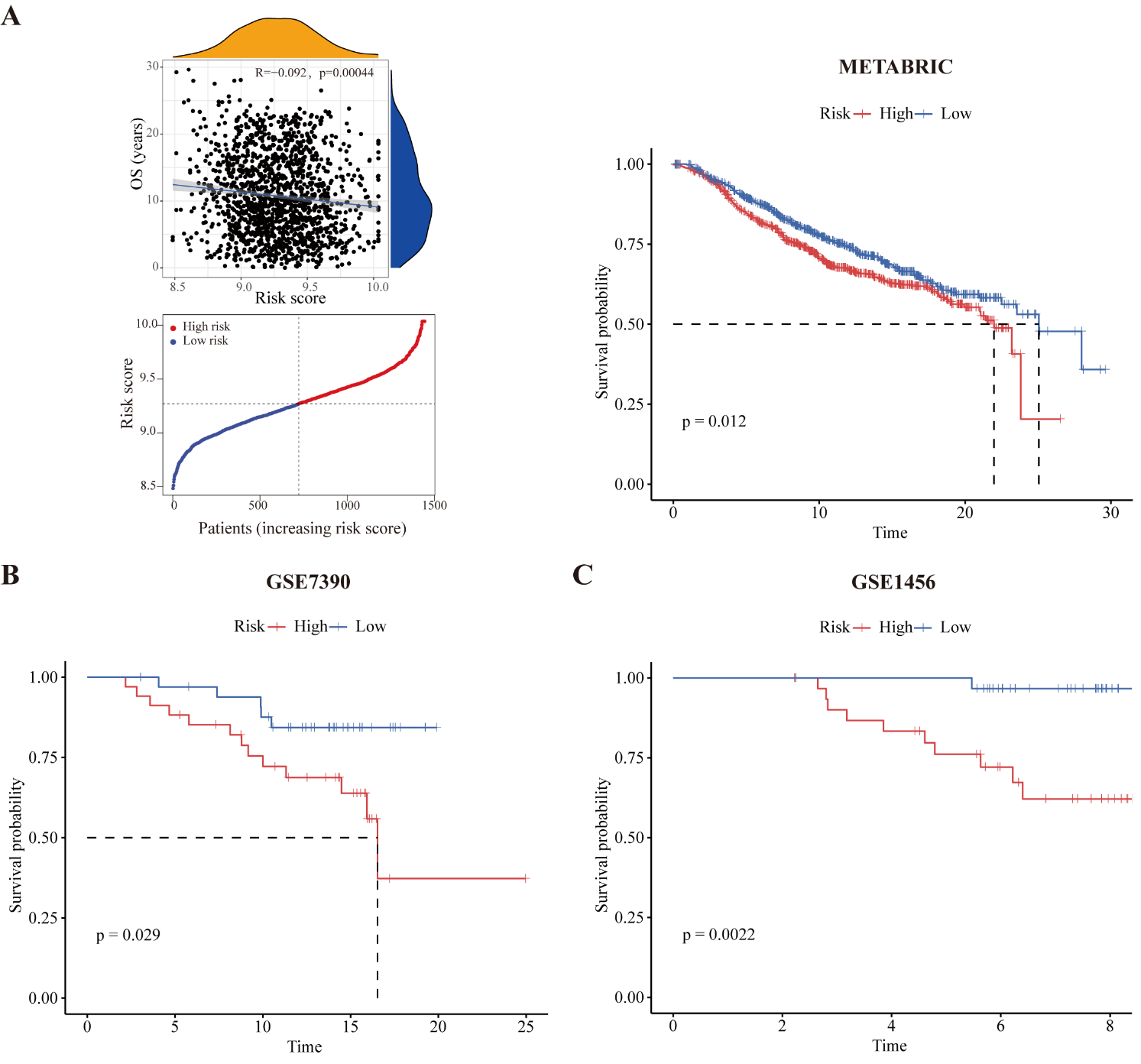
**

**Supplementary Figure 5.** Prognostic value validation of LMRG-based signature in the METABRIC cohort (A), GSE7390 cohort (B) and GSE1456 cohort (C).

**
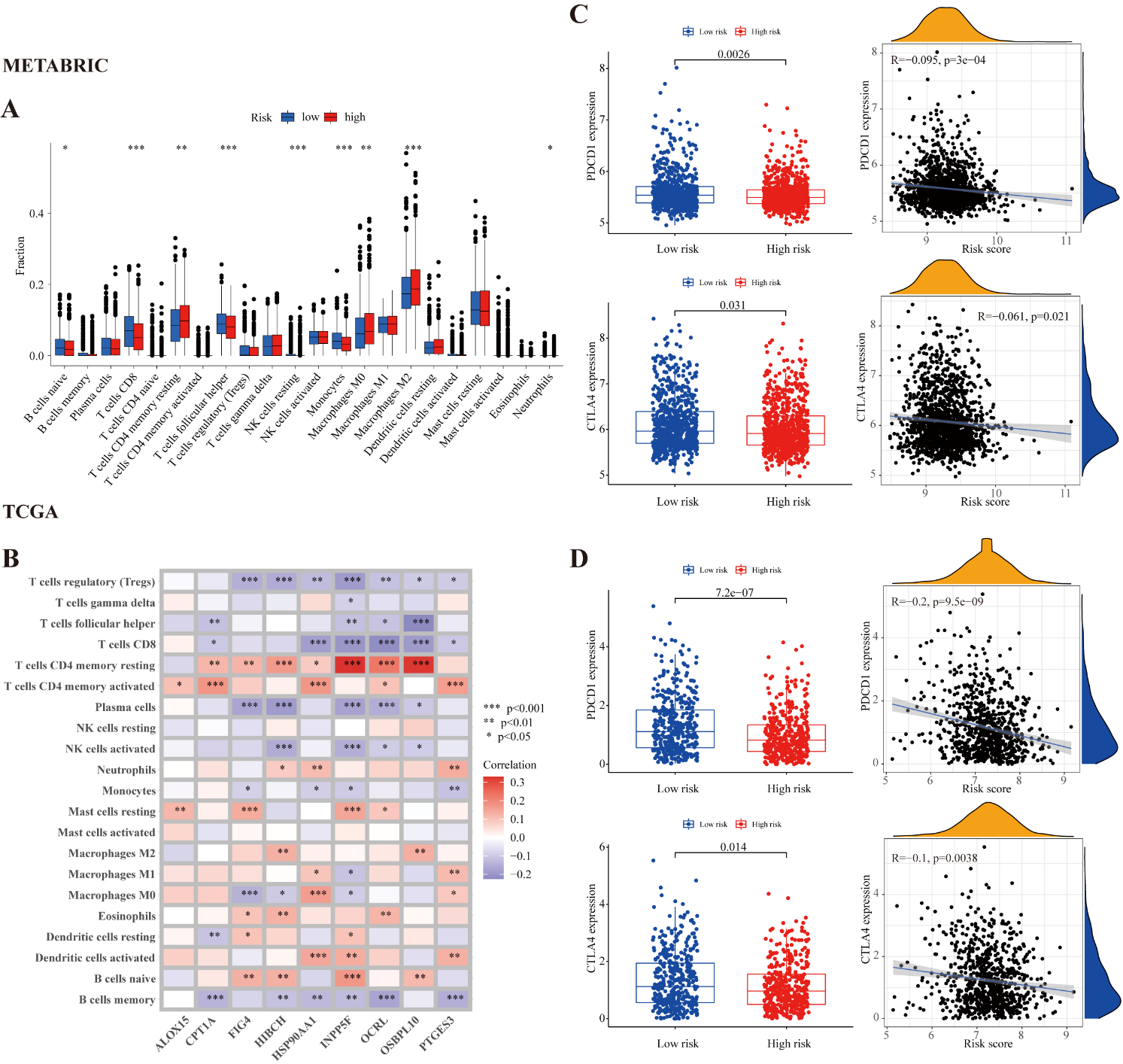
**

**Supplementary Figure 6.** (A) Immune cell infiltration levels of the two risk groups in the METABRIC cohort. (B) Correlation between immune cell infiltration and expression of nine LMRGs in the cohort from TCGA. (C, D) Estimated PDCD1 and CTLA4 levels in the two risk groups from METABRIC are shown. *p < 0.05, **p < 0.01, ***p < 0.001.

**
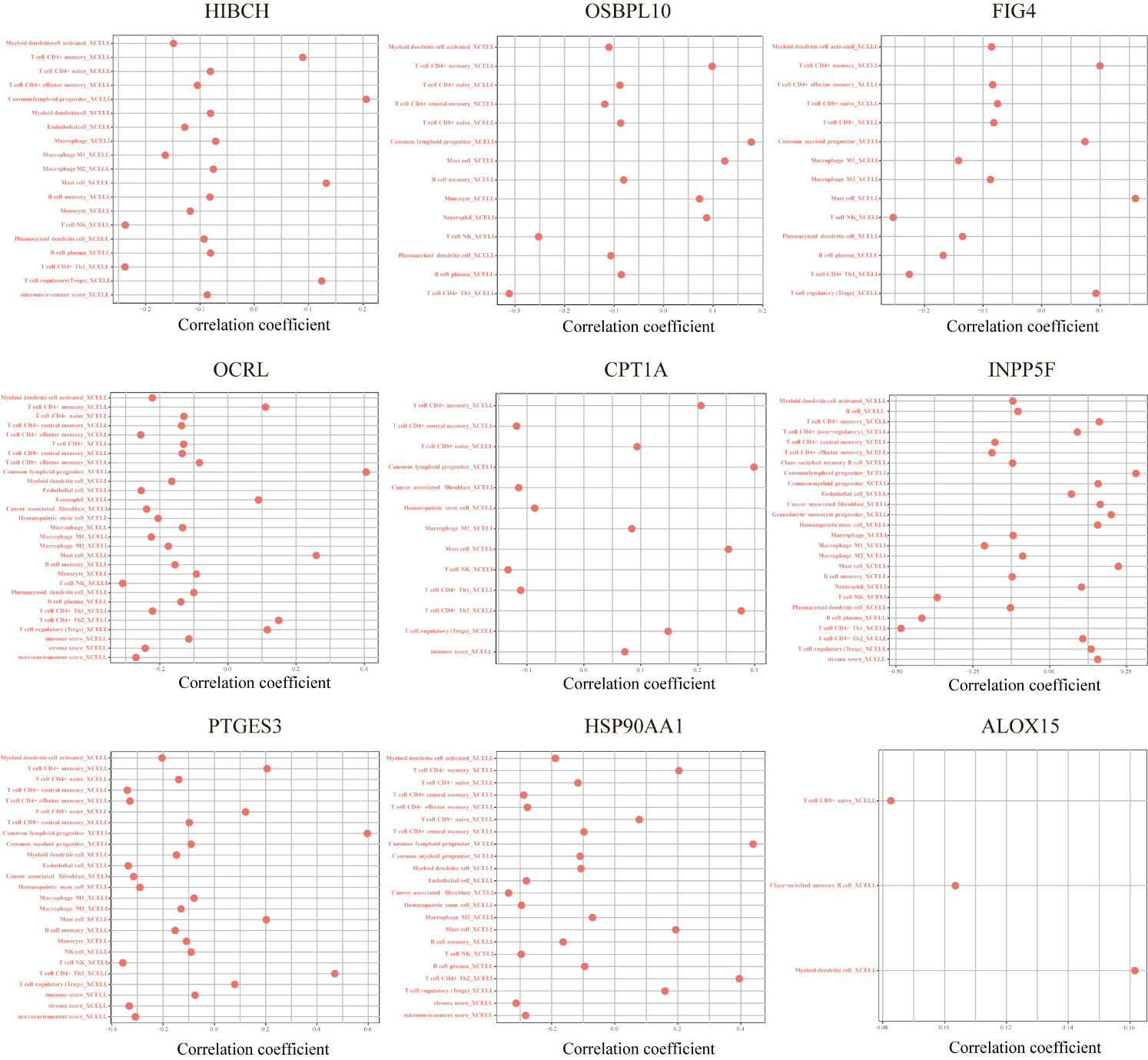
**

**Supplementary Figure 7.** Correlation between immune cell infiltration and expression of nine LMRGs in cohort from TCGA according to the xCell algorithm.

**
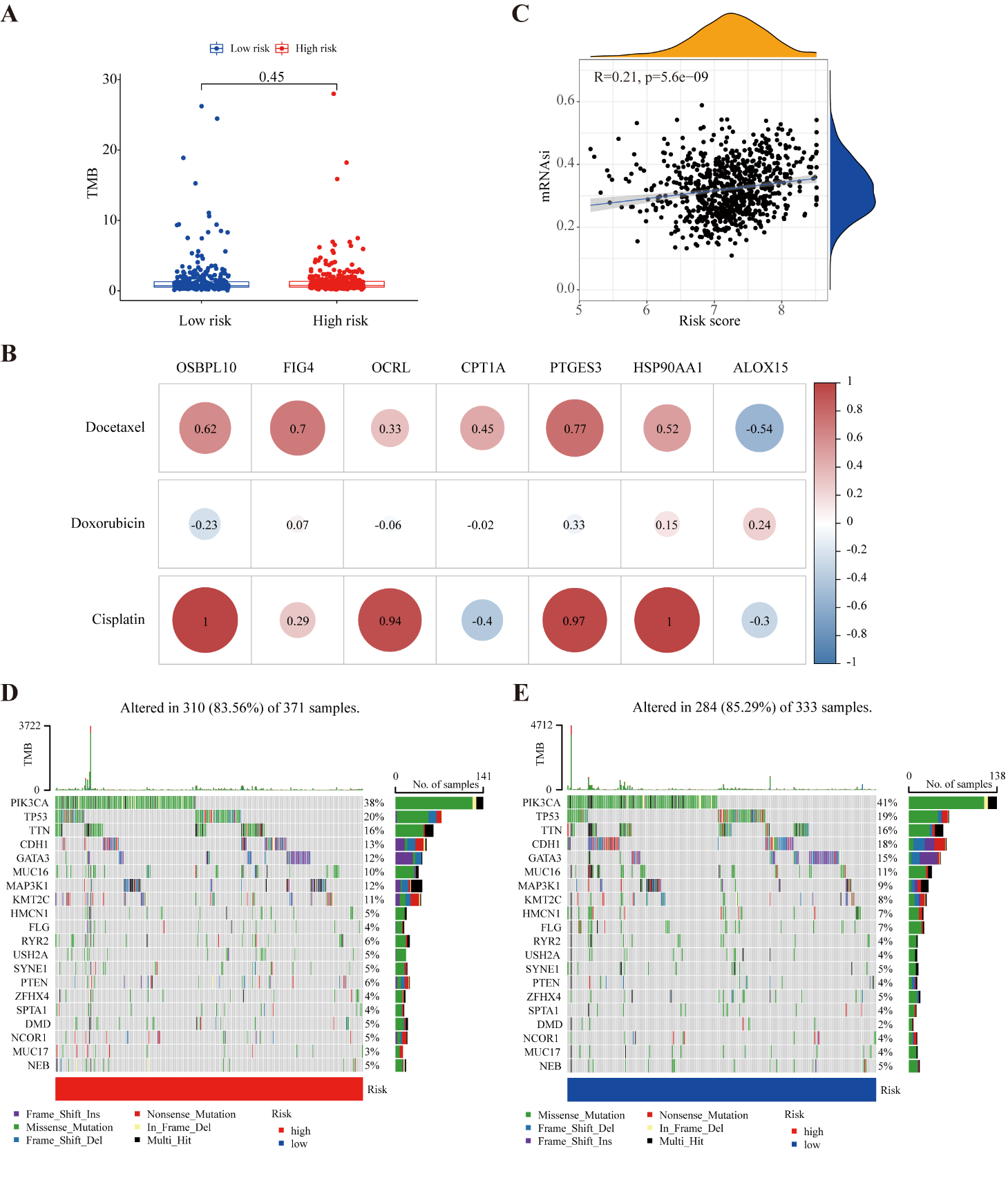
**

**Supplementary Figure 8.** TMB (A), stemness features (C) and top mutated genes (D-E) in high- and low-risk groups in the cohort from TCGA. (B) Correlations between specific gene expression and IC50 values of docetaxel, doxorubicin and cisplatin in BC cell lines using the CCLE database.

**
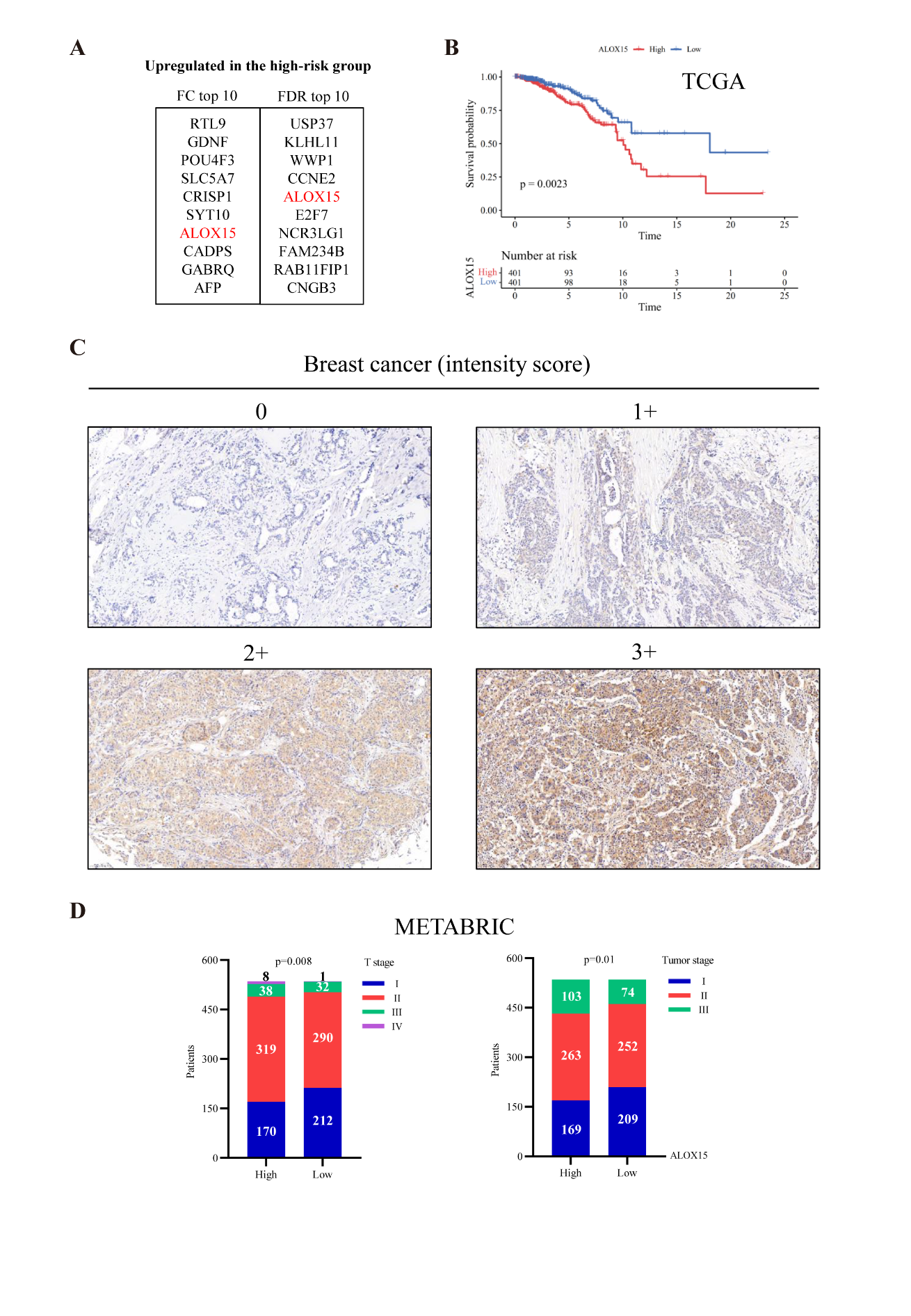
**

**Supplementary Figure 9.** (A) List of genes upregulated in the high-risk group ordered by FC and FDR. (B) K-M survival curves stratified by ALOX15 mRNA expression in TCGA. (C) Specific immunostaining of the ALOX15 protein in primary tumors of ER+ BC was confirmed. Intensity was scored according to the degree of staining (0–3). (D) ALOX15 expression was associated with T stage and tumor stage in the METABRIC database. Comparisons were performed using the chi-square test.

## Supplementary Tables

Supplementary Table 1: Clinical characteristics of the patients in TCGA, METABRIC and GEO datasets.

Supplementary Table 2: List of lipid metabolism-associated genes from the GSEA database.

Supplementary Table 3: Name of the candidate immune checkpoints.

Supplementary Table 4: Univariate Cox regression analysis results for identifying LMRGs associated with OS of ER + BC.

Supplementary Table 5: Log-rank test results for selecting candidate LMRGs.

Supplementary Table 6: The prognostic values of LMRG CpG sites in BC patients by MethSurv platform.

Supplementary Table 7: DEGs between the two risk groups from TCGA-ER+ BC.

Supplementary Table 8: List of 91 genes belonging to six clusters of metagenes.
