# Supplementary material for: Exploration of prognosis and immunometabolism landscapes in ER+ breast cancer based on a novel lipid metabolism-related signature
Source: Front Immunol. 2023 Jul 4;14:1199465. doi: 10.3389/fimmu.2023.1199465 (PMC10352658; doi:10.3389/fimmu.2023.1199465)
Supplement: Supplementary file 2 [file Table_1.pdf]

Characteristic	TCGA n=802	METABRIC n=1444	GSE7390 n=134	GSE25066 n=298	GSE4779 n=40
Age					
<=60	419	540	134	243	
>60	383	904		55	
T stage					
I	211	530	76	18	
II	452	823	58	150	29
III	111	71		82	11
IV	26	9		45	
N stage					
N0	354	782	134	104	16
N1	283	444		141	24
N2	90	156		35	
N3	59	62		18	
Stage					
I	139	453	76	2	
II	442	739	58	169	34
III	190	243		126	6
IV	15				
Grade					
I		156	29	29	
II		675	68	152	
III		556	35	101	
PR status					
Positive	676	945		224	25
Negative	123	499		73	15
