# Supplementary material for: Exploration of prognosis and immunometabolism landscapes in ER+ breast cancer based on a novel lipid metabolism-related signature
Source: Front Immunol. 2023 Jul 4;14:1199465. doi: 10.3389/fimmu.2023.1199465 (PMC10352658; doi:10.3389/fimmu.2023.1199465)
Supplement: Supplementary file 4 [file Table_3.pdf]

IDO1
LAG3
CTLA4
TNFRSF9
ICOS
CD80
PDCD1LG2
TIGIT
CD70
TNFSF9
ICOSLG
KIR3DL1
CD86
PDCD1
LAIR1
TNFRSF8
TNFSF15
TNFRSF14
IDO2
CD276
CD40
TNFRSF4
TNFSF14
HHLA2
CD244
CD274
HAVCR2
CD27
BTLA
LGALS9
TMIGD2
CD28
CD48
TNFRSF25
CD40LG
ADORA2A
VTCN1
CD160
CD44
TNFSF18
TNFRSF18
BTNL2
C10ORF54
CD200R1
TNFSF4
CD200
NRP1
