# Supplementary material for: Exploration of prognosis and immunometabolism landscapes in ER+ breast cancer based on a novel lipid metabolism-related signature
Source: Front Immunol. 2023 Jul 4;14:1199465. doi: 10.3389/fimmu.2023.1199465 (PMC10352658; doi:10.3389/fimmu.2023.1199465)
Supplement: Supplementary file 5 [file Table_4.pdf]

id	HR	HR.95L	HR.95H	pvalue
FABP7	0.796247	0.715804	0.88573	2.75E-05
ALDH3A1	0.823972	0.751443	0.903501	3.81E-05
CA6	0.867991	0.805327	0.935531	0.000213
CYP24A1	0.880552	0.819689	0.945935	0.0005
ALOX15	1.156189	1.064803	1.255417	0.000551
OCRL	1.583378	1.213332	2.066281	0.000715
CERS3	0.882821	0.821111	0.94917	0.000749
NFKBIE	0.676905	0.534054	0.857967	0.001253
CYP27A1	0.772061	0.657381	0.906747	0.001615
ACBD5	1.474306	1.155115	1.881699	0.001819
CYP2D6	0.77761	0.660354	0.915686	0.00256
SOAT2	0.890336	0.825353	0.960435	0.002665
CEL	1.177693	1.05804	1.310878	0.002771
NCOA2	1.404911	1.124196	1.755723	0.002796
CBR1	0.784433	0.668673	0.920233	0.002879
THEM5	0.756365	0.628548	0.910175	0.003112
PTGES3	1.639032	1.180549	2.275571	0.003163
PCYT2	0.686489	0.533731	0.882967	0.003399
CBR3	0.785705	0.6683	0.923736	0.003493
ADH6	0.886235	0.816826	0.961542	0.003703
S1PR4	0.836061	0.740759	0.943623	0.003735
CHKB	0.687521	0.533578	0.885878	0.003769
SYNJ2	1.462226	1.129548	1.892886	0.003916
NFKBIA	0.660259	0.497602	0.876085	0.004018
CDK19	1.349904	1.099673	1.657076	0.004127
OSBPL10	1.4121	1.113348	1.791018	0.004437
ACAA1	0.677111	0.517272	0.886341	0.004537
SPHK1	0.769603	0.641839	0.922799	0.004693
CYP4B1	1.140785	1.040749	1.250436	0.004909
INSIG2	1.525931	1.135937	2.049818	0.00501
PRKAB2	1.463591	1.119196	1.913963	0.005392
HSP90AA1	1.393485	1.098737	1.767303	0.006209
HACD3	1.368414	1.08981	1.718241	0.006925
CPT1A	1.309823	1.075168	1.59569	0.007373
MBTPS2	1.373802	1.088722	1.733531	0.007444
PLA2G2D	0.910532	0.849869	0.975525	0.007713
BLVRA	0.746433	0.601856	0.92574	0.007758
GPX4	0.704881	0.54434	0.91277	0.007999
FAM120B	1.487111	1.104844	2.001639	0.008854
SERINC1	1.450122	1.096436	1.917898	0.009178
LIPH	1.228569	1.050879	1.436304	0.009807
BCHE	1.106271	1.024478	1.194594	0.009965
CCNC	1.403618	1.084254	1.81705	0.01005
ST3GAL1	1.267472	1.056893	1.520008	0.010562
PLPPR1	0.935698	0.889137	0.984697	0.010707
CLOCK	1.371293	1.073866	1.751097	0.011365
LIPG	0.854322	0.756254	0.965106	0.011378
CPNE3	1.329076	1.062999	1.661753	0.012561
POMC	0.869903	0.778783	0.971685	0.013558
GSTZ1	0.734537	0.574827	0.938619	0.013649
GPX1	0.733242	0.571561	0.94066	0.014635
STAR	0.898639	0.824569	0.979362	0.014887
HACD1	0.831262	0.716279	0.964703	0.014972
NR1H3	0.732582	0.569428	0.942484	0.015487
CYP4F12	0.908685	0.840593	0.982293	0.015974
SLCO1A2	0.93699	0.888601	0.988013	0.016141
MTM1	1.417765	1.066791	1.88421	0.01615

SEC24A	1.32303	1.050849	1.665708	0.017218
FIG4	1.348232	1.054078	1.724474	0.017343
UGT2B4	1.066709	1.011338	1.12511	0.017572
DGKZ	0.701612	0.523277	0.940725	0.017868
MMUT	1.410104	1.058647	1.878239	0.018793
PLA2G12A	1.370864	1.051187	1.787757	0.019888
CYP4F11	0.9135	0.845905	0.986496	0.021077
ALDH2	0.842413	0.727601	0.97534	0.021792
CHUK	1.366935	1.046323	1.785788	0.021905
APOA5	1.050659	1.00698	1.096233	0.022545
FDX2	0.742246	0.574495	0.958978	0.022583
LPAR3	0.916073	0.849259	0.988143	0.023288
FUT6	0.9328	0.878217	0.990775	0.023745
TSPO	0.770905	0.615157	0.966086	0.023846
PTGES	0.857142	0.749689	0.979997	0.024094
SMARCD3	0.787074	0.639183	0.969185	0.024151
CPTP	0.749	0.582483	0.963121	0.024268
GRHPR	0.688838	0.49795	0.952903	0.024364
CERS6	1.234061	1.025255	1.485393	0.02617
ALOX15B	0.904364	0.827601	0.988247	0.026337
B3GALT4	0.778768	0.624536	0.971087	0.026384
PRKCQ	0.878519	0.782521	0.986295	0.028257
SYNJ1	1.303822	1.028603	1.65268	0.028301
FUT7	0.901041	0.820848	0.989069	0.028447
MTMR9	1.337646	1.031127	1.735284	0.028466
B3GNT2	1.342858	1.031067	1.748934	0.028748
ACADVL	0.728502	0.548447	0.967669	0.028753
TBL1X	1.302631	1.027773	1.650994	0.028778
DGKE	1.219805	1.02049	1.458049	0.029051
INPP5F	1.34615	1.030201	1.758996	0.029408
ENPP6	0.869318	0.766342	0.986132	0.029477
MOGAT2	0.918277	0.850415	0.991555	0.02952
PIK3CD	0.813612	0.675309	0.98024	0.030013
PLD4	0.860493	0.750804	0.986207	0.030804
AHRR	1.18191	1.015462	1.375642	0.030921
AGPAT5	1.296144	1.02362	1.641224	0.031256
MED23	1.335117	1.025853	1.737614	0.031569
HSD17B6	1.232273	1.018326	1.49117	0.031825
G0S2	0.893775	0.806115	0.990967	0.032987
HPGD	0.90267	0.82154	0.991812	0.033083
B3GALT1	0.919492	0.85114	0.993333	0.033197
CYP1B1	0.853655	0.737722	0.987808	0.033612
HIBCH	1.376342	1.024713	1.848631	0.033825
SOCS3	0.831459	0.700964	0.986247	0.034095
PRXL2B	0.750642	0.575205	0.979588	0.034703
PLA2G2A	0.929145	0.867814	0.99481	0.034917
TRADD	0.762706	0.591406	0.983621	0.036869
APOD	0.90638	0.826158	0.994391	0.037624
PLEKHA3	1.416036	1.019678	1.966462	0.037869
FITM1	0.868242	0.759703	0.992287	0.038116
ACAD11	1.273296	1.011962	1.602118	0.039263
ELOVL6	1.20931	1.008974	1.449424	0.039721
B3GNT3	0.916267	0.842881	0.996044	0.04007
NFKBIB	0.754336	0.575925	0.988015	0.040606
CYP7B1	0.859583	0.743373	0.99396	0.04118
SLC10A1	1.077008	1.002779	1.156732	0.041737
LTC4S	0.958435	0.920053	0.998419	0.041768
MAPK9	1.350304	1.011131	1.803248	0.041854

PTPRG	1.242201	1.00694	1.53243	0.042917
LPAR2	0.747974	0.564509	0.991064	0.043127
NBN	1.276514	1.007256	1.617751	0.043406
FABP5	0.847873	0.722066	0.995599	0.044032
SLC27A6	0.93077	0.867598	0.998543	0.045432
MMP1	1.08039	1.001501	1.165493	0.045638
APOA1	0.931623	0.869049	0.998703	0.045875
CIDEC	0.941805	0.887806	0.999088	0.046563
RAB5A	1.389684	1.004824	1.921951	0.046695
SEC24C	1.235612	1.003054	1.522089	0.046743
PLEKHA4	0.853843	0.730091	0.998573	0.047946
S1PR5	0.856891	0.735192	0.998735	0.048136
GPS2	0.786811	0.620135	0.998284	0.04837
FUT2	0.880698	0.776277	0.999164	0.048501
STARD3NL	1.406268	1.001846	1.973946	0.048767
PPT1	1.281348	0.999976	1.641891	0.050022
GLUL	0.822355	0.676148	1.000177	0.050208
PTGS2	0.897627	0.805642	1.000114	0.050242
GSTM4	0.806903	0.650514	1.00089	0.050954
CYP11B1	1.039277	0.999817	1.080294	0.051093
HSD11B1	0.890485	0.792061	1.00114	0.05227
LCLAT1	1.285648	0.996607	1.658519	0.053137
PIK3C2G	0.928578	0.861356	1.001045	0.053271
PPARG	0.855641	0.729334	1.003821	0.055728
TXNRD1	1.22239	0.994927	1.501855	0.055934
SLC44A1	1.297622	0.991523	1.69822	0.057703
PTPN13	1.162535	0.99427	1.359277	0.059035
PIP4K2C	1.266572	0.990842	1.619032	0.059225
SAMD8	1.235332	0.990592	1.540539	0.060648
GGT5	0.839646	0.699014	1.00857	0.061662
MTMR1	1.349474	0.985294	1.848261	0.061812
CYP3A43	0.956595	0.912921	1.002359	0.062721
VAPB	1.260284	0.987606	1.608248	0.062927
MED13	1.203441	0.989974	1.462937	0.063053
ACSM3	0.836549	0.692873	1.010017	0.063412
MED14	1.258937	0.986612	1.606429	0.064086
ERP29	0.765111	0.575317	1.017519	0.065684
SLC44A3	0.838782	0.695271	1.011915	0.066321
PCYT1A	1.333253	0.98057	1.812786	0.066536
STK11	0.783999	0.603876	1.01785	0.067681
RAB14	1.343835	0.977918	1.846672	0.068413
GPX6	1.057781	0.995728	1.1237	0.06858
ACOT9	0.816783	0.656907	1.01557	0.068614
AGPAT1	1.40455	0.974323	2.024752	0.068674
ELOVL2	0.934697	0.868952	1.005416	0.069553
HCCS	1.345401	0.976077	1.854468	0.069974
AGMO	0.924816	0.849842	1.006405	0.069994
A4GALT	0.841635	0.698309	1.014377	0.070281
UROD	0.734857	0.526319	1.026022	0.070433
SAR1B	1.311017	0.97728	1.758723	0.070818
ACER2	1.134383	0.988757	1.301457	0.072071
SUCLA2	1.293004	0.977181	1.710901	0.072112
SDHC	1.300137	0.97666	1.730753	0.07215
OXCT1	1.186244	0.984444	1.429412	0.072627
MTMR12	1.238187	0.980262	1.563978	0.073023
OLAH	0.93543	0.869419	1.006453	0.073827
MVD	0.826743	0.670863	1.018842	0.074285
S1PR2	0.796684	0.620603	1.022723	0.074481

SPTLC1	1.290459	0.974514	1.708835	0.075112
ADH1C	0.941972	0.881934	1.006097	0.075229
CIDEA	0.951526	0.90056	1.005377	0.076889
ACOXL	1.111404	0.988645	1.249407	0.07694
CPNE6	0.963882	0.925289	1.004085	0.07766
STARD10	0.861711	0.730298	1.016771	0.077907
CHD9	1.213016	0.978501	1.503735	0.078121
AUH	1.325371	0.96747	1.815671	0.079424
SUMF1	1.295678	0.969863	1.730947	0.07962
IL4I1	0.886276	0.774232	1.014534	0.079995
RDH16	1.103354	0.988115	1.232034	0.080546
ACSS3	1.142943	0.982635	1.329405	0.083137
HSPH1	1.198348	0.976518	1.470569	0.083193
GAD2	1.03486	0.995473	1.075806	0.083493
PNPLA2	0.808267	0.634534	1.029568	0.084714
TGS1	1.257755	0.968907	1.632713	0.084945
GPX5	1.057708	0.99204	1.127724	0.08624
SULT1E1	0.949789	0.895409	1.007471	0.086801
PIK3CA	1.239143	0.969174	1.584313	0.087225
NCOA3	1.207197	0.972718	1.498198	0.087465
FASN	0.886124	0.770968	1.01848	0.088726
PIK3CB	1.246365	0.966913	1.606583	0.089092
ACADL	0.946359	0.88806	1.008487	0.089231
MED17	1.299337	0.959836	1.758923	0.090139
ETNPPL	0.957486	0.910511	1.006885	0.090528
PMVK	0.812941	0.638791	1.034568	0.092244
PIK3R4	1.284242	0.959334	1.719189	0.092763
PRKAG1	1.367266	0.949318	1.969221	0.092852
LYPLA1	1.202926	0.969667	1.492297	0.092977
HSD3B7	0.839313	0.683863	1.030099	0.0937
CDS1	1.227257	0.964622	1.561399	0.095556
GLA	0.84165	0.687101	1.030962	0.095835
ACSL5	0.879117	0.755088	1.023519	0.096839
GDPD5	0.849945	0.701411	1.029933	0.097114
HSDL2	1.253535	0.959322	1.63798	0.097786
UGT1A8	0.909306	0.812363	1.017818	0.098347
FABP4	0.944654	0.882944	1.010678	0.098569
CYP4F22	0.939429	0.872193	1.011849	0.099131
SLC33A1	1.255659	0.957357	1.64691	0.099957
ACOT1	0.868936	0.73464	1.02778	0.10099
PLA2G4B	0.915389	0.823588	1.017422	0.101082
PNPLA3	0.928918	0.850546	1.014511	0.101086
PRKD2	0.777686	0.573963	1.053719	0.104731
PIKFYVE	1.221267	0.958555	1.55598	0.105781
ADH7	0.942118	0.876441	1.012716	0.105827
FITM2	1.207234	0.96062	1.517159	0.106232
OSBPL6	1.135165	0.973033	1.324313	0.106897
PDPK1	1.253623	0.952385	1.650141	0.106953
BCKDHB	1.225887	0.956056	1.571874	0.108346
TNFRSF1B	0.863428	0.721484	1.033297	0.109042
CSNK2A1	1.276683	0.946771	1.721556	0.109295
PIK3C2A	1.207824	0.958374	1.522203	0.109656
CD1D	0.869511	0.732354	1.032354	0.110395
PSME1	0.804636	0.616026	1.050994	0.110712
MIGA2	0.787455	0.586507	1.057251	0.111923
UGT1A6	1.046232	0.98949	1.106228	0.112156
ECI1	0.822498	0.646126	1.047015	0.112542
MBOAT7	1.228641	0.952437	1.584943	0.112994

B3GALNT1	1.185723	0.960387	1.46393	0.11317
CERS2	1.222914	0.953364	1.568675	0.113186
MED11	0.777152	0.56887	1.061693	0.113221
PRKAA2	0.93215	0.854259	1.017144	0.114532
IRS4	0.933166	0.856122	1.017144	0.115641
SLCO1B1	1.03614	0.991305	1.083002	0.115717
TECRL	1.050525	0.987782	1.117252	0.116713
CA2	0.922952	0.834933	1.020249	0.116896
ABCC3	0.885868	0.761335	1.030771	0.116913
PTPN11	1.208549	0.953566	1.531715	0.117184
ALDH1A1	0.893649	0.776161	1.02892	0.117928
MED15	0.761718	0.540694	1.073091	0.11958
CYP27B1	0.894006	0.776331	1.029517	0.119711
HSD17B8	0.847673	0.687436	1.04526	0.12213
FUT9	1.053469	0.986027	1.125523	0.122805
ABO	0.922951	0.833493	1.022009	0.12321
PPP1CA	0.807549	0.615301	1.059862	0.12335
PLB1	0.865885	0.720794	1.040182	0.123817
FAR1	1.202305	0.950251	1.521217	0.124819
PTGDS	0.92225	0.83168	1.022684	0.124865
ESYT1	1.270397	0.934933	1.72623	0.126046
LEP	0.955963	0.902219	1.012909	0.127132
CYP8B1	0.943893	0.876364	1.016625	0.127354
CERS1	0.929354	0.845768	1.021201	0.127592
INPP4B	1.129028	0.965348	1.32046	0.128853
TIAM2	1.187546	0.950966	1.482982	0.129414
FABP6	1.045986	0.986867	1.108647	0.129871
TNFAIP8	0.843383	0.676436	1.051533	0.130158
CDK8	1.19844	0.946034	1.518191	0.133563
PLPPR2	1.238854	0.936265	1.639238	0.133862
STARD5	0.839767	0.667845	1.055947	0.135129
PTGES2	0.807565	0.609703	1.069637	0.136093
GK3P	1.032265	0.990004	1.076329	0.136504
MED12	1.261723	0.928875	1.713843	0.136807
AKR1C3	0.890154	0.763573	1.03772	0.137055
MED1	1.132768	0.960816	1.335494	0.137781
CYP4A22	1.043186	0.986438	1.103199	0.138471
HSD17B2	0.946748	0.880447	1.018042	0.139608
MED7	1.305161	0.916364	1.858918	0.139962
B4GALT2	0.791219	0.579062	1.081108	0.141475
SLC44A2	1.2438	0.929581	1.664231	0.141977
SEC23A	1.196092	0.941291	1.519866	0.142931
ST6GALNAC	1.156144	0.952002	1.40406	0.143267
ADIPOR2	1.155172	0.952196	1.401416	0.143436
PITPNM1	0.865221	0.712649	1.050456	0.143565
MED18	1.305122	0.913223	1.865201	0.143824
ADIPOQ	0.960681	0.910371	1.013771	0.143852
GLIPR1	0.853704	0.690024	1.05621	0.145279
PI4K2B	1.213656	0.934562	1.576097	0.146402
ADH1B	0.95939	0.906995	1.014813	0.147952
APOC3	1.033096	0.988426	1.079785	0.148805
EBP	0.82686	0.638726	1.070408	0.148908
ARSF	0.964659	0.91866	1.012961	0.148919
PIK3C3	1.230467	0.927935	1.631633	0.149732
PLD2	0.805695	0.599948	1.082001	0.150975
GPD1L	1.163042	0.946357	1.42934	0.151051
ECH1	0.819693	0.623509	1.077605	0.154307
HDAC3	1.307957	0.903409	1.893664	0.155044

NFYA	1.214681	0.928808	1.588542	0.155454
SEC24B	1.220345	0.926758	1.606936	0.15612
ANGPTL4	0.918178	0.815886	1.033295	0.156636
GPAT2	0.913136	0.804765	1.0361	0.158605
MAOA	0.922981	0.825436	1.032052	0.159616
CYP3A7	1.127292	0.953741	1.332423	0.16011
DPEP1	0.921235	0.821525	1.033046	0.160409
ACSL1	1.117892	0.956768	1.306151	0.160489
PISD	0.839861	0.65813	1.071774	0.160677
ACLY	1.188232	0.933162	1.513022	0.161851
CBR4	1.204547	0.927489	1.564368	0.16286
SEC24D	1.160338	0.941498	1.430046	0.16313
RORA	1.175898	0.934374	1.479853	0.167185
NCAPH2	0.818903	0.616399	1.087935	0.168061
BAAT	1.037631	0.98452	1.093607	0.168209
CYP4F2	0.963621	0.914136	1.015784	0.168286
HSD17B3	1.069908	0.971546	1.178229	0.169658
TNFRSF1A	0.802292	0.585791	1.098809	0.169827
SQLE	1.120346	0.95247	1.317811	0.170059
NCOA6	1.213229	0.918819	1.601974	0.1729
SGPP1	0.890562	0.753767	1.052182	0.173149
PLA2G4F	1.09868	0.959073	1.258608	0.174693
ACSF2	0.886381	0.744697	1.055021	0.174707
DBI	0.846504	0.665353	1.076975	0.174981
NCOR1	1.182944	0.927871	1.508136	0.175157
GDE1	1.241104	0.907712	1.696947	0.175958
TSPOAP1	0.898421	0.769117	1.049463	0.176684
ACBD7	1.082012	0.964646	1.213658	0.178452
PHYH	1.171772	0.929712	1.476856	0.179383
JAK2	0.859333	0.688421	1.072678	0.180283
HSD17B4	1.210853	0.915125	1.602148	0.18052
TPTE2	1.044777	0.979856	1.113999	0.180816
IRS2	0.899813	0.770852	1.05035	0.181038
DPEP3	0.966775	0.920056	1.015866	0.181205
ESRRA	0.848633	0.666599	1.080378	0.182739
SIN3B	0.806149	0.586973	1.107166	0.183155
HACD2	1.168059	0.929126	1.468436	0.183388
FDXR	0.867717	0.704016	1.069482	0.183449
TNFAIP8L1	0.831112	0.632292	1.09245	0.184805
DGKA	0.858942	0.685712	1.075933	0.185799
GGPS1	1.227806	0.905443	1.664941	0.186592
UGT2B10	1.031627	0.984965	1.080499	0.187337
FAAH2	1.173567	0.92439	1.489912	0.188737
HADHB	1.272234	0.887101	1.824571	0.190608
NUDT19	1.159052	0.928316	1.447139	0.192501
LHB	0.964051	0.91232	1.018716	0.193255
AKR1D1	0.959859	0.902408	1.020968	0.193256
CSNK1G2	0.818833	0.605751	1.106871	0.193701
MTMR2	1.177715	0.920251	1.507211	0.193723
LCAT	0.872529	0.71025	1.071886	0.194024
ACHE	0.919328	0.809728	1.043764	0.194067
SGMS2	1.106239	0.94979	1.288459	0.194355
CYP2C9	1.028385	0.98582	1.072788	0.194357
ACSL6	0.924723	0.821606	1.040781	0.19451
OXCT2	0.958454	0.898927	1.021923	0.194608
GPX7	0.885772	0.737044	1.064512	0.195888
GDPD1	1.121158	0.94254	1.333625	0.196492
CPNE7	0.933805	0.841403	1.036355	0.197658

GPD1	0.952278	0.883965	1.02587	0.197928
UGT1A1	1.048892	0.975239	1.128106	0.198789
SELENOI	1.160398	0.924784	1.456041	0.198902
GAL3ST1	0.951574	0.881931	1.026717	0.200531
SP1	1.228415	0.894269	1.687416	0.20406
CYP2A13	1.03361	0.982098	1.087823	0.205009
ACER3	1.139774	0.929819	1.397138	0.207861
DECR2	0.857463	0.674921	1.089377	0.208008
LRP2	1.043956	0.976313	1.116286	0.208174
PLPP2	1.141933	0.928591	1.40429	0.208454
MTMR8	0.926243	0.821552	1.044275	0.210563
DHRS7B	0.845327	0.649776	1.09973	0.210654
ETFDH	1.195978	0.902248	1.585332	0.213281
PPARGC1A	0.927326	0.823007	1.044868	0.215294
GPCPD1	0.862138	0.681621	1.090463	0.215902
LPCAT1	1.156729	0.917962	1.4576	0.217093
CPT1B	0.887682	0.734532	1.072763	0.217559
UGT8	0.94101	0.854149	1.036703	0.218513
CYP2C18	1.027189	0.984214	1.072041	0.218602
GPAM	1.122798	0.933564	1.350389	0.218716
AWAT2	0.96898	0.921271	1.01916	0.221243
APOA2	1.030304	0.982111	1.080861	0.221922
LYPLA2	0.829315	0.613484	1.121079	0.223659
GDPD3	1.077316	0.955495	1.214668	0.22384
SBF2	1.13209	0.926604	1.383145	0.224737
NDUFAB1	1.221907	0.884041	1.688901	0.224898
RAP1GDS1	1.14598	0.919488	1.428262	0.225191
DHCR24	1.132398	0.92621	1.384486	0.22533
KPNB1	1.191974	0.897319	1.583386	0.225461
PLIN1	0.951084	0.87688	1.031568	0.226251
PCCA	1.155418	0.914347	1.460047	0.226291
ACOX2	1.06582	0.960971	1.182108	0.227634
OSBP	1.188561	0.897453	1.574097	0.228147
OSBPL2	1.186469	0.897991	1.56762	0.22899
MTF1	1.164689	0.907322	1.495059	0.231463
SRD5A2	0.943523	0.857222	1.038513	0.234905
MTMR10	1.187196	0.893085	1.578163	0.237427
PIP5K1B	0.936731	0.840365	1.044146	0.237991
GLB1	1.195259	0.888601	1.607744	0.238335
ARF3	1.20252	0.884528	1.634831	0.239229
AKR1C1	0.942628	0.854107	1.040323	0.240282
INPP4A	1.196435	0.885868	1.615882	0.242151
PIK3C2B	1.174971	0.896148	1.540545	0.243362
DECR1	1.171808	0.895315	1.533688	0.248233
AGPAT3	1.188345	0.885574	1.594631	0.250113
PLA2G1B	0.966955	0.913118	1.023966	0.250276
B4GALT6	1.104281	0.932275	1.308022	0.250879
PCK1	0.966894	0.912866	1.024119	0.25114
PLPPR3	0.960116	0.895443	1.02946	0.252652
DGKB	1.043303	0.970135	1.121989	0.253172
PLEKHA6	1.136719	0.911958	1.416875	0.254266
MAPK10	1.080448	0.945505	1.23465	0.255648
HACD4	1.099736	0.93332	1.295826	0.256103
SMS	1.18347	0.884493	1.583507	0.256872
S1PR1	0.90593	0.763617	1.074764	0.257195
FDPS	0.850914	0.643552	1.125091	0.257261
IDI2	1.022184	0.984097	1.061746	0.257418
PLA2G2C	0.964398	0.905674	1.026929	0.258073

AWAT1	0.974083	0.930613	1.019585	0.259616
ME1	1.073065	0.948977	1.213378	0.260714
ACOX1	1.172989	0.887393	1.5505	0.262383
HAO2	0.95883	0.890832	1.032018	0.262628
COMT	0.859848	0.660208	1.119858	0.262635
MIGA1	1.127384	0.913844	1.390823	0.263111
GBGT1	0.882643	0.709189	1.098522	0.263464
EP300	1.151541	0.899163	1.474758	0.263621
PLPPR4	1.09387	0.934043	1.281045	0.265578
SORBS1	0.915585	0.783641	1.069746	0.266662
PTEN	1.175816	0.88274	1.566196	0.268177
SUCLG2	1.166784	0.887419	1.534096	0.269317
DDHD1	1.165298	0.887841	1.529462	0.270218
ACSL4	0.908119	0.764815	1.078274	0.271371
MED26	0.827027	0.589187	1.160875	0.272321
PLPPR5	1.038867	0.97039	1.112177	0.273072
CPOX	1.166894	0.884613	1.53925	0.274703
SLC2A1	1.121251	0.912962	1.37706	0.275058
PTPMT1	0.851346	0.637652	1.136655	0.275118
PTGR1	1.137111	0.901839	1.433759	0.277304
MOGAT3	1.02093	0.983421	1.05987	0.278095
BMX	0.945779	0.855066	1.046115	0.278535
PLIN3	0.840787	0.614248	1.150873	0.278955
TRAF2	0.853861	0.641335	1.136813	0.27931
CERS4	1.121528	0.911091	1.380571	0.279364
PON1	0.963907	0.901749	1.03035	0.279762
PECR	1.129058	0.905803	1.407339	0.280214
DEGS2	1.074713	0.94236	1.225655	0.282565
MVK	0.85699	0.64607	1.136769	0.284327
CYP21A2	0.947381	0.858002	1.046071	0.285021
SLC25A1	0.871164	0.674827	1.124624	0.289801
METAP1	1.177601	0.869563	1.594761	0.290685
TMEM86B	0.896971	0.733122	1.097441	0.290739
LEPR	1.082832	0.934068	1.255289	0.291243
ST8SIA1	0.9257	0.801862	1.068664	0.292044
ACER1	1.037985	0.96821	1.112788	0.293699
PRKD1	1.099122	0.921153	1.311477	0.294326
ARSI	0.933416	0.820174	1.062293	0.296393
LPAR1	1.121669	0.904038	1.391691	0.296814
B4GALT1	0.888098	0.71057	1.109981	0.29697
LSS	1.14744	0.885942	1.486124	0.297303
APEX1	0.842811	0.610786	1.162979	0.297906
SC5D	1.117799	0.906327	1.378614	0.297986
GBA2	0.844055	0.613014	1.162174	0.298827
NAGA	0.852566	0.630191	1.153409	0.300949
CHPT1	0.918179	0.780947	1.079526	0.301367
CYP4F8	0.971549	0.919757	1.026257	0.301755
SBF1	0.872026	0.672374	1.130962	0.301956
PRKAG3	0.973984	0.926254	1.024175	0.303853
SMPD1	1.165393	0.87012	1.560865	0.304554
SLC25A17	1.169164	0.865643	1.579108	0.308142
HMGCR	1.136658	0.888254	1.454528	0.308624
GLYCTK	0.880617	0.689437	1.124812	0.308638
ASAH2	1.071214	0.937848	1.223547	0.310549
UGT2A1	0.96588	0.903136	1.032983	0.311045
RXRG	1.049578	0.9557	1.152677	0.311461
INPP5D	0.911889	0.76228	1.090861	0.313071
MCAT	0.862048	0.64558	1.1511	0.314335

PLA2G4A	0.927009	0.799412	1.074972	0.315798
TNFAIP8L2	0.907453	0.750169	1.097713	0.317318
UGT2A3	1.020565	0.980634	1.062121	0.317483
ST3GAL2	0.877529	0.679178	1.133806	0.317623
SLC2A4	0.939681	0.831407	1.062054	0.319215
PPM1L	1.111222	0.90218	1.3687	0.321281
NR1D1	1.102297	0.909111	1.336535	0.32183
ACACA	1.114691	0.899124	1.381942	0.322071
GC	1.030661	0.970612	1.094427	0.324106
LPGAT1	1.125428	0.889807	1.42344	0.324195
FABP12	0.95686	0.876128	1.045031	0.326807
ADIPOR1	1.156608	0.864686	1.547084	0.326923
FUT4	1.114265	0.897237	1.383788	0.327633
INPP5J	1.076032	0.928411	1.247126	0.330385
ACSF3	0.865063	0.645838	1.158702	0.330997
PNLIP	0.975304	0.926731	1.026423	0.337366
DGKQ	0.864284	0.641351	1.164708	0.337938
CDIPT	1.169334	0.848994	1.610545	0.338199
SLC27A2	0.956523	0.873278	1.047703	0.338646
CYP4F3	0.9688	0.907748	1.033959	0.339879
PIP4K2B	1.130882	0.87744	1.457529	0.342082
ALAD	1.16047	0.850582	1.583259	0.347759
OSBPL8	1.095053	0.905704	1.323988	0.34853
STS	0.916218	0.762491	1.100938	0.350423
ARNT2	1.066223	0.931814	1.220021	0.350969
MAPKAPK2	0.884027	0.682277	1.145434	0.351008
OLR1	1.076572	0.921642	1.257546	0.35202
PTS	1.161476	0.846198	1.594222	0.35423
GPD2	1.099127	0.899872	1.342503	0.354365
IDI1	1.116205	0.884094	1.409254	0.355355
FAAH	0.898841	0.716874	1.126996	0.35545
PI4KA	1.127796	0.873195	1.45663	0.356914
PLAAT3	1.084861	0.912241	1.290147	0.356958
LCN12	0.945953	0.840122	1.065116	0.358693
DLD	1.140814	0.860231	1.512916	0.360362
STARD7	1.150264	0.851693	1.553502	0.361237
MTMR3	0.878755	0.665394	1.160531	0.362389
PLPP6	1.133306	0.865078	1.484702	0.363801
ACAT1	1.119556	0.877261	1.428772	0.364101
PLEKHA5	1.130665	0.866853	1.474765	0.36498
PEX11A	1.106374	0.888822	1.377176	0.365508
ABCA1	1.094811	0.899697	1.332239	0.365722
AQP7	0.96538	0.894187	1.04224	0.367344
MBOAT2	1.083477	0.909787	1.290328	0.368448
GLB1L	1.12523	0.870008	1.455322	0.368668
AKR1B1	0.906677	0.731831	1.123297	0.370106
ST6GALNAC	1.047802	0.945993	1.160568	0.370592
UBC	0.85315	0.602598	1.207878	0.370628
UGCG	0.926865	0.78478	1.094674	0.371036
MED20	1.149252	0.847264	1.558875	0.371123
DPEP2	0.921921	0.770887	1.102547	0.373169
PRDX6	1.149706	0.845209	1.563903	0.374175
SLC22A5	1.117758	0.874291	1.429026	0.374452
CYP46A1	1.092059	0.899146	1.326361	0.374543
MMAA	1.155034	0.840255	1.587735	0.374633
LPL	0.948641	0.844208	1.065992	0.375598
CRLS1	1.163944	0.831906	1.628509	0.375638
AKR1C2	0.959522	0.875536	1.051564	0.376623

NCOR2	0.881734	0.666418	1.166617	0.378251
RAB4A	1.14253	0.846073	1.542863	0.384644
HSD3B1	0.981329	0.940381	1.02406	0.386113
AGT	0.960537	0.876937	1.052106	0.386137
ARV1	1.128759	0.858264	1.484504	0.386216
PLA2G2F	1.020806	0.974172	1.069672	0.388053
PIP5K1A	1.133914	0.851176	1.510569	0.39044
SACM1L	1.135275	0.848877	1.5183	0.392345
MCEE	1.137919	0.846238	1.530137	0.392519
AKT2	0.868113	0.627585	1.200825	0.392882
PON3	1.032624	0.959132	1.111748	0.394072
HSD3B2	1.018562	0.976257	1.062699	0.395467
PIK3R2	0.901113	0.708497	1.146094	0.396089
CEPT1	1.117579	0.864332	1.445028	0.396494
SULT2B1	1.056627	0.930105	1.200359	0.397292
VAPA	1.151195	0.83082	1.59511	0.397471
HMGCS1	1.096896	0.885192	1.359232	0.397932
SPTSSA	1.119345	0.861219	1.454836	0.399269
RUFY1	1.164937	0.815156	1.664808	0.402
SREBF2	0.906505	0.720296	1.140851	0.402755
ARSB	1.099884	0.879287	1.375826	0.404512
SLC44A5	1.038028	0.950652	1.133435	0.405453
LIPE	0.947766	0.835072	1.07567	0.406198
FUT5	0.981688	0.939717	1.025533	0.407086
FDFT1	1.116958	0.858996	1.452388	0.409058
ALAS1	1.143885	0.831259	1.574085	0.409189
AGPAT2	0.914911	0.740441	1.130491	0.410064
HSD17B14	0.928955	0.778524	1.108452	0.413575
AKT1	0.879055	0.645208	1.197656	0.413969
ACOT13	1.128445	0.844283	1.508248	0.414274
MOGAT1	0.982243	0.940659	1.025665	0.416916
PCCB	1.123422	0.847132	1.489825	0.419053
INMT	0.953041	0.847824	1.071316	0.420343
TECR	0.886609	0.66107	1.189095	0.421643
ACP6	0.908468	0.718728	1.148298	0.421914
CYP3A4	0.975484	0.917993	1.036576	0.423197
MTMR6	1.107219	0.862514	1.421351	0.424126
ALDH7A1	1.113874	0.854895	1.451306	0.424426
PLA2G3	1.021428	0.969641	1.07598	0.424495
B3GALT5	0.970065	0.900176	1.045379	0.425647
SPTLC2	1.102126	0.867425	1.40033	0.426097
SCD5	0.942569	0.814288	1.091059	0.428125
SPTLC3	1.066173	0.90974	1.249504	0.428665
SREBF1	0.931772	0.782131	1.110041	0.428846
PLAAT2	1.045664	0.935496	1.168806	0.431814
ABCD1	1.106167	0.859698	1.423297	0.432726
LBR	0.921579	0.751199	1.130601	0.433608
PI4K2A	1.157944	0.801465	1.672978	0.434732
LIPF	0.978088	0.925045	1.034172	0.436082
ARNTL	1.112667	0.849213	1.457853	0.438699
GCNT2	0.949902	0.833935	1.081995	0.43912
B3GALT2	1.052365	0.924641	1.197733	0.439436
ALPI	0.978397	0.925618	1.034185	0.440163
CYP2U1	1.082897	0.884345	1.326027	0.440923
TDO2	1.045875	0.932694	1.17279	0.442739
ADPRM	0.879721	0.633464	1.22171	0.444375
ORMDL3	1.070655	0.898653	1.275578	0.444833
SDHA	1.129714	0.825903	1.545284	0.445382

CREBBP	1.119919	0.836822	1.498788	0.4462
PITPNM3	1.066593	0.903081	1.259712	0.44767
TP53INP2	1.086589	0.876851	1.346494	0.447893
CROT	0.929385	0.768842	1.123452	0.449124
PNPLA7	0.927701	0.763601	1.127068	0.449897
CTSA	1.11835	0.835012	1.497831	0.453031
PLTP	0.930413	0.770254	1.123873	0.454256
ARF1	1.128725	0.821457	1.550926	0.45514
TNF	0.952509	0.838226	1.082374	0.455595
RELA	0.882187	0.634662	1.226249	0.455634
LGALS1	0.916889	0.729935	1.151728	0.455795
CYP39A1	0.960817	0.86489	1.067385	0.456383
PLBD1	0.938421	0.793672	1.10957	0.45714
PLA2G6	0.919124	0.73565	1.148358	0.457891
INPP5K	0.885114	0.641252	1.221714	0.457993
MED21	1.114858	0.836217	1.486346	0.458705
AMACR	1.101232	0.853223	1.421332	0.458879
LIPA	1.085762	0.873314	1.349892	0.458909
CYP3A5	1.071373	0.892184	1.286552	0.460348
HEXA	0.889656	0.651121	1.215577	0.46285
NFYB	1.126866	0.818211	1.551956	0.464544
ARSK	1.097559	0.85502	1.408899	0.465009
PPARGC1B	0.937283	0.787047	1.116195	0.467424
CYP2F1	0.980412	0.929335	1.034296	0.468647
YWHAH	0.893726	0.659141	1.211797	0.469499
DGAT2	0.954291	0.840537	1.083441	0.470016
AKR1A1	0.886799	0.639826	1.229104	0.470695
NTHL1	0.922621	0.74107	1.148651	0.471304
UROS	0.886669	0.639156	1.23003	0.471374
UGDH	0.939557	0.792859	1.113398	0.471643
ARSJ	1.056088	0.910139	1.22544	0.472053
HEXB	1.122251	0.819161	1.537484	0.472716
ACSBG1	0.941056	0.796507	1.111837	0.475219
ANKRD1	0.970983	0.895501	1.052828	0.475744
CA4	0.980425	0.928445	1.035314	0.476897
ORMDL1	1.123263	0.814717	1.548658	0.478083
S100A10	0.927947	0.754622	1.141082	0.478396
PIK3R6	0.934341	0.774296	1.127468	0.478671
MDH1	1.135095	0.799061	1.612443	0.479252
SCD	1.046876	0.921889	1.188807	0.480067
GK2	1.035953	0.939056	1.142849	0.480823
ALB	1.027641	0.952468	1.108748	0.481752
GRHL1	1.063521	0.894842	1.263996	0.484581
MTMR4	1.090236	0.854447	1.391093	0.487155
MID1IP1	1.085175	0.861598	1.366769	0.487414
FUT3	0.973178	0.900846	1.051318	0.490216
ACADM	0.922327	0.732995	1.160562	0.490357
GGT1	1.041323	0.927294	1.169373	0.493787
GBA	1.091285	0.849495	1.401894	0.494245
PPP1CC	1.114941	0.814558	1.526095	0.496935
FHL2	1.052711	0.907663	1.220939	0.497059
LCN15	1.013821	0.974354	1.054887	0.49806
SIN3A	1.10855	0.822519	1.494047	0.498537
PTGR2	1.095285	0.84077	1.426846	0.499965
LTA4H	0.899338	0.660326	1.224862	0.500866
TRIB3	1.074361	0.871862	1.323894	0.500872
FABP1	1.015537	0.970939	1.062182	0.501039
HADH	0.908675	0.687074	1.201748	0.501932

NEU2	0.965428	0.870792	1.07035	0.503877
GALC	1.088294	0.848052	1.396593	0.506134
ARSA	0.923013	0.728635	1.169246	0.506692
CARM1	1.103472	0.824973	1.475988	0.50703
CDS2	1.102228	0.825973	1.47088	0.508492
AGPAT4	0.934405	0.763254	1.143934	0.51102
PTDSS2	0.90953	0.68486	1.207905	0.512412
PLEKHA8	1.084353	0.850413	1.382647	0.513665
BDH1	1.082067	0.853034	1.372594	0.515694
ADH1A	0.985462	0.94279	1.030066	0.516737
NRF1	0.872404	0.57683	1.319432	0.517828
ALOX12B	0.981051	0.925739	1.039667	0.518186
RETSAT	1.098274	0.825578	1.461044	0.519753
ACBD4	0.9325	0.753209	1.154468	0.521204
PRKAG2	0.904604	0.665903	1.228869	0.521246
ARSD	1.079552	0.854351	1.364114	0.521358
SLC27A1	0.916676	0.701506	1.197843	0.523863
IDH3G	0.898542	0.645512	1.250755	0.526083
REEP6	0.963288	0.85809	1.081383	0.526138
GPAT4	1.06967	0.868419	1.317559	0.526526
NEU1	1.092658	0.829896	1.438616	0.527777
CAV1	0.946038	0.796254	1.123997	0.528179
TM7SF2	0.948814	0.805518	1.117601	0.529359
NR1H2	0.898235	0.642865	1.255047	0.529443
SRD5A3	1.078982	0.851308	1.367544	0.52957
SLC10A2	1.020639	0.957175	1.088312	0.532824
ESYT2	1.092637	0.826592	1.444311	0.533756
PGS1	0.918362	0.702227	1.201019	0.533907
BMPR1B	1.018768	0.960399	1.080684	0.536792
PLA2G4D	0.97974	0.918089	1.04553	0.537069
HSD17B7	0.927068	0.728362	1.179983	0.538363
NPAS2	0.953811	0.820318	1.109029	0.538733
TNFRSF21	1.057798	0.884057	1.265684	0.539351
CERK	1.077668	0.848475	1.368771	0.539801
ACOX3	1.073697	0.854782	1.348676	0.541054
GPX3	0.957733	0.833833	1.100043	0.541207
RXRB	0.899057	0.638549	1.265843	0.542155
GABARAPL1	1.077626	0.846265	1.37224	0.544327
MIF	0.945819	0.789934	1.132467	0.544384
PSAP	1.085256	0.832357	1.414994	0.545571
HSD17B1	0.946461	0.791563	1.131669	0.546208
PEMT	0.925659	0.719983	1.190089	0.54681
FABP9	1.015199	0.966544	1.066304	0.547185
MGLL	1.052727	0.890394	1.244656	0.547606
MED31	1.107299	0.794109	1.544007	0.547921
ILK	1.078215	0.840184	1.383682	0.55404
ORMDL2	1.09037	0.818501	1.452541	0.55435
ENO3	0.940657	0.767295	1.153189	0.556124
DDHD2	1.052333	0.88685	1.248693	0.55898
CSNK2B	0.909468	0.661454	1.250476	0.559148
PLD3	0.914769	0.678256	1.233757	0.55945
DGKH	1.0553	0.880723	1.264483	0.559643
INPP5E	0.917008	0.683399	1.230471	0.563593
PNPLA8	1.086954	0.815934	1.447995	0.56881
PIP4K2A	1.084858	0.819671	1.43584	0.569003
CH25H	0.961934	0.841446	1.099675	0.569767
ACOT11	1.047	0.891848	1.229143	0.574626
LPAR5	1.05787	0.868185	1.288998	0.576856

SUMF2	0.922754	0.694879	1.225359	0.578524
PPP1CB	1.087667	0.808295	1.463599	0.579017
SCAP	0.911824	0.657921	1.263713	0.579336
FH	1.08499	0.813097	1.4478	0.579437
AHR	0.947646	0.783174	1.146657	0.580335
AOC3	0.95812	0.822421	1.116209	0.582965
PLA2G10	0.971856	0.87735	1.076541	0.584418
CYP2A7	1.019198	0.951936	1.091211	0.585137
LIPC	1.036072	0.91176	1.177334	0.586853
HMGCL	0.918703	0.67501	1.250376	0.589775
CHKA	0.928105	0.707559	1.217396	0.589912
CYP4A11	0.983683	0.926312	1.044607	0.591548
VNN1	0.958891	0.822164	1.118355	0.592771
ST3GAL4	0.937734	0.740709	1.187165	0.593176
LDHA	1.070591	0.833465	1.375181	0.593365
FDX1	0.921577	0.682424	1.244541	0.594183
ABHD5	1.07294	0.827679	1.390878	0.594952
OSBPL3	0.952769	0.796421	1.13981	0.596776
KMT5A	0.920237	0.676067	1.252591	0.597235
PNPLA6	0.920861	0.678005	1.250706	0.597629
RAN	1.080838	0.808524	1.444866	0.599672
SUCLG1	1.100488	0.76965	1.573539	0.599683
SULT2A1	1.010502	0.971812	1.050733	0.599934
MED9	0.924106	0.68701	1.243026	0.60182
OSBPL9	0.926418	0.694547	1.235698	0.60305
ARNT	1.083037	0.801286	1.463858	0.603839
CYP2B6	1.013677	0.962195	1.067913	0.609483
ACBD6	0.918015	0.661003	1.274959	0.609741
NSDHL	1.080959	0.800715	1.459286	0.611149
CAMKK2	0.918176	0.660461	1.276454	0.611555
D2HGDH	0.945026	0.758555	1.177334	0.614116
NR1H4	0.985581	0.930575	1.043838	0.620109
CYP2C19	1.014756	0.957637	1.075283	0.6202
ADH4	0.988247	0.943057	1.035602	0.62055
B4GALT4	1.07233	0.812381	1.415459	0.621998
JMJD7.PLA2(	0.957213	0.803114	1.14088	0.625349
ALDH9A1	1.072719	0.808764	1.422821	0.626176
FUT1	1.0501	0.862555	1.278423	0.626258
MORC2	1.079028	0.794223	1.465962	0.626641
UGT1A10	0.981365	0.909726	1.058646	0.626699
LPCAT2	0.95499	0.793077	1.14996	0.627063
THRSP	1.017804	0.947716	1.093075	0.62783
CYP1A2	1.009254	0.972115	1.047813	0.630123
ECI2	1.06041	0.834539	1.347412	0.631265
PNPLA4	1.064724	0.822892	1.377626	0.633296
PITPNM2	0.956048	0.794657	1.150217	0.633753
ALOX5	0.960193	0.812275	1.135048	0.634151
MBTPS1	1.070467	0.80753	1.419019	0.635861
CGA	0.985697	0.928541	1.046371	0.636438
PLAAT4	0.969245	0.851239	1.10361	0.637209
ABHD3	1.047731	0.861069	1.274859	0.64138
PTGIS	0.968025	0.843628	1.110765	0.643313
MED16	0.934207	0.699879	1.246992	0.64416
UGT2B28	0.986093	0.929041	1.046647	0.645096
B3GNT5	0.966884	0.837767	1.115902	0.645175
DGAT1	1.063674	0.816884	1.385022	0.646733
ADH5	1.083592	0.768375	1.528122	0.647148
TBXAS1	0.949534	0.76034	1.185803	0.64784

ACSBG2	1.035096	0.892534	1.200428	0.648221
MECR	0.922929	0.65357	1.303301	0.64875
PPARA	0.958159	0.796673	1.152378	0.649915
IDH1	0.949596	0.759214	1.187717	0.650524
EHHADH	1.049296	0.852036	1.292225	0.650627
ST8SIA5	1.028082	0.911951	1.159001	0.650657
ACSL3	1.054818	0.836584	1.329981	0.651806
ESYT3	0.970033	0.848797	1.108586	0.655132
PDHB	0.932446	0.685458	1.268429	0.655964
FMO1	0.973293	0.863814	1.096647	0.656588
ST3GAL5	1.046996	0.854473	1.282897	0.65778
PIK3R1	0.957683	0.79089	1.159652	0.657864
DHCR7	0.960587	0.803794	1.147964	0.658298
SDHD	1.069103	0.792735	1.441819	0.661472
SLCO1B3	0.98456	0.917916	1.056043	0.663475
AKR1B15	0.979144	0.889824	1.077431	0.665852
MSMO1	1.056807	0.822089	1.358541	0.666342
GNPAT	1.074237	0.775192	1.488644	0.667051
MTOR	1.066254	0.79533	1.429466	0.667989
ACAD10	1.068517	0.788717	1.447577	0.668793
ASAH1	1.039974	0.868332	1.245544	0.670184
PLA2G5	1.031861	0.891969	1.193694	0.673065
PLEKHA1	1.05526	0.820805	1.356684	0.674797
LPIN1	0.955758	0.772181	1.182978	0.67754
CPNE1	1.053802	0.822898	1.349496	0.677936
ST6GALNAC	1.040152	0.862294	1.254695	0.680742
THRAP3	1.074988	0.761593	1.517346	0.680919
CAMKK1	1.052391	0.824488	1.343288	0.681743
ALDH3B2	1.01955	0.928513	1.119513	0.684947
ALDH1B1	1.051715	0.824117	1.342167	0.685297
MED13L	1.040831	0.857077	1.263982	0.686359
LPCAT3	1.041796	0.853578	1.271516	0.687136
HSD17B10	0.942873	0.707653	1.256279	0.68787
ETNK1	1.049349	0.828938	1.328365	0.688846
ACADS	0.963118	0.800861	1.158249	0.689721
VDR	1.051504	0.821694	1.345588	0.689787
PNPLA5	1.009006	0.965514	1.054457	0.690026
CYP2J2	0.972087	0.84535	1.117825	0.691218
STARD4	0.958452	0.7773	1.181824	0.691358
B4GALT3	1.05656	0.801086	1.393508	0.696857
PTDSS1	1.050252	0.820223	1.344793	0.697475
ADSL	0.938609	0.681343	1.293015	0.698277
ABCC1	1.056829	0.798974	1.397903	0.698519
MED4	1.070463	0.75812	1.511489	0.698886
PCK2	0.949244	0.726467	1.240337	0.702686
THEM4	1.052716	0.808409	1.370855	0.702967
DLST	1.066138	0.766011	1.483856	0.704187
GAPDHS	1.007704	0.968437	1.048563	0.705098
ACOT6	1.012693	0.948416	1.081325	0.706186
MED29	0.937098	0.668054	1.314494	0.706724
FABP2	1.007287	0.969433	1.04662	0.710255
ODC1	0.965888	0.801844	1.163494	0.71477
ABCB11	1.009555	0.959326	1.062414	0.714944
HTD2	1.011804	0.948739	1.079061	0.720809
PLA2G4C	0.964178	0.789338	1.177745	0.720832
AKR1C4	0.99239	0.951429	1.035114	0.722422
LCN9	1.011211	0.950867	1.075384	0.722502
PDHA1	1.059965	0.768549	1.461879	0.722562

SPTSSB	1.01501	0.93485	1.102043	0.72263
NCOA1	1.047611	0.809882	1.355121	0.723193
GGT7	0.962825	0.780107	1.188339	0.72421
ACOT8	1.051147	0.791961	1.395158	0.729858
HSD11B2	1.029199	0.873876	1.212128	0.730241
UGT2B7	1.010597	0.951705	1.073133	0.730761
PLIN2	0.967975	0.804122	1.165216	0.730853
ALOX5AP	0.970166	0.816024	1.153425	0.731528
SLC27A3	0.958382	0.751207	1.222694	0.7323
ECHS1	0.946874	0.692428	1.29482	0.732445
PPARD	0.949515	0.705508	1.277915	0.732481
UGT2B11	0.99095	0.940549	1.044052	0.732832
ELOVL5	1.029063	0.872018	1.214389	0.734548
LIPI	0.98835	0.923006	1.05832	0.73703
SLC25A20	1.044947	0.805441	1.355672	0.740638
ST3GAL6	0.964401	0.777597	1.196082	0.741414
ARSH	1.017852	0.915876	1.131182	0.742525
PRKACA	1.060519	0.746486	1.506661	0.74293
PLEKHA2	0.966386	0.786962	1.186718	0.744207
ALOXE3	1.017873	0.914753	1.132617	0.745143
CUBN	0.965308	0.779835	1.194893	0.745683
PITPNB	1.05153	0.775544	1.425728	0.746327
GGT6	1.015871	0.921569	1.119824	0.751403
ACOT4	0.972702	0.81901	1.155236	0.752443
ALOX12	0.967197	0.785405	1.191066	0.753534
ELOVL1	0.949697	0.687278	1.312314	0.754438
ABCB4	1.035715	0.829358	1.293418	0.756906
AGK	1.056315	0.74622	1.495272	0.75733
HPGDS	0.97436	0.825218	1.150457	0.759276
BDH2	1.038587	0.815207	1.323178	0.759285
RGL1	1.029237	0.855797	1.237828	0.75955
ELOVL7	0.978177	0.848987	1.127025	0.760131
HSD17B13	0.989067	0.919911	1.063422	0.766277
LPIN2	1.034927	0.825005	1.298264	0.766606
PHOSPHO1	0.975672	0.828019	1.149655	0.768623
AACS	0.962381	0.743644	1.245457	0.77069
SLC44A4	1.016437	0.910712	1.134435	0.771106
HSD17B12	1.037317	0.80966	1.328987	0.771961
CPT1C	0.967767	0.774965	1.208535	0.772553
NEU4	1.008459	0.952243	1.067993	0.773473
SGPP2	0.985773	0.893709	1.08732	0.774535
ACAT2	1.033719	0.823494	1.29761	0.774973
MLYCD	1.041741	0.78641	1.379971	0.775602
HADHA	1.050785	0.744823	1.482433	0.777847
SMPD4	0.956327	0.698773	1.308809	0.780293
IKBKG	1.03848	0.796402	1.354141	0.780376
ABHD4	1.042974	0.77576	1.402231	0.780537
HMGCLL1	0.989363	0.916379	1.06816	0.784456
PCBD1	1.036781	0.798237	1.346611	0.786576
CHAT	0.989934	0.919971	1.065219	0.786764
SCP2	1.041721	0.773958	1.402122	0.787439
UGT1A9	1.008497	0.94787	1.073001	0.789109
PPT2	1.042723	0.767294	1.41702	0.789214
ETNK2	0.979083	0.837872	1.144092	0.790228
ALDOA	1.032778	0.81243	1.312887	0.792234
OSBPL1A	0.970765	0.775515	1.215175	0.795662
LGMN	1.031957	0.81316	1.309624	0.795834
HILPDA	1.027853	0.831219	1.271004	0.799815

MTMR7	0.980904	0.844866	1.138846	0.800175
IKBKB	1.030719	0.814794	1.303865	0.800837
PRKACB	1.016886	0.891961	1.159307	0.802292
TKFC	1.036018	0.78464	1.367931	0.80294
CRYZ	0.973552	0.786493	1.205101	0.805513
UBE2I	1.048874	0.71398	1.540852	0.80788
S1PR3	0.977711	0.813717	1.174757	0.809847
GCDH	1.036044	0.773971	1.386856	0.811899
MED19	1.037205	0.76131	1.413083	0.816908
PIAS4	0.961891	0.689019	1.34283	0.819451
ACOT2	0.973457	0.772079	1.227361	0.820039
LPIN3	0.973362	0.769583	1.2311	0.82177
NEU3	1.02717	0.812757	1.298147	0.822439
PTGS1	0.979393	0.816001	1.175501	0.823068
RXRA	0.968551	0.730522	1.284139	0.824274
HACL1	1.033417	0.772022	1.383316	0.825147
STARD3	0.979375	0.813818	1.178613	0.825422
LPCAT4	1.031663	0.77684	1.370074	0.829489
ACAA2	1.027052	0.805295	1.309875	0.829702
FADS1	0.983754	0.847052	1.142518	0.8301
G6PC2	1.004488	0.963874	1.046813	0.831614
INSIG1	0.978061	0.795772	1.202108	0.833049
CYP11A1	0.987402	0.8775	1.111068	0.833212
UBE2L6	0.979614	0.807763	1.188027	0.834231
EPHX2	1.017535	0.864457	1.19772	0.834469
IDH3B	0.966622	0.700021	1.334758	0.836645
ELOVL4	1.015397	0.877958	1.174353	0.836853
MED28	1.033261	0.752071	1.419584	0.840001
HMGCS2	0.994558	0.941756	1.050319	0.844556
MED24	0.978535	0.785548	1.218933	0.846489
ACOT12	0.996227	0.958483	1.035458	0.847896
AGRP	1.008134	0.92733	1.095978	0.849278
TBL1XR1	1.025121	0.793632	1.324131	0.849315
PLAAT5	1.007806	0.92976	1.092403	0.850042
UCP1	1.00594	0.945938	1.069748	0.850302
CYP19A1	1.008808	0.920809	1.105217	0.850625
GPX2	0.990972	0.901494	1.089333	0.851019
GLTP	1.02884	0.756502	1.399218	0.856185
PLA2G12B	1.003507	0.965713	1.042781	0.858141
STAT3	0.976547	0.750517	1.27065	0.859755
PIP4P1	0.969553	0.686657	1.368999	0.860563
ST6GALNAC	0.973782	0.717682	1.321271	0.86451
CPT2	0.976262	0.738376	1.290788	0.866109
PLA2G4E	0.995266	0.94175	1.051823	0.866355
ALDH3B1	1.018615	0.81905	1.266805	0.86833
CYP2E1	0.988565	0.862712	1.132777	0.868518
AKT3	1.013237	0.866417	1.184936	0.86922
PLPP1	0.980442	0.773509	1.242736	0.870285
IRS1	1.012972	0.865208	1.185971	0.872718
PLA1A	1.012234	0.872024	1.174988	0.873
ENO2	1.012138	0.872158	1.174584	0.873778
LCN1	1.003542	0.959971	1.049091	0.875934
STARD6	0.995979	0.946717	1.047805	0.876301
AADAT	1.012032	0.869991	1.177264	0.876819
PRKAA1	1.017847	0.812241	1.275501	0.877882
PLA2G15	1.0215	0.776997	1.342941	0.87888
PIP5K1C	0.971692	0.670216	1.408777	0.879558
ACO2	0.98124	0.766969	1.255373	0.880246

AGPS	1.0126	0.859275	1.193283	0.881186
UGT1A4	1.007766	0.909356	1.116826	0.882695
CYP17A1	0.988262	0.844615	1.156341	0.882868
FAR2	1.011308	0.869266	1.176559	0.884229
CD36	1.006714	0.919125	1.102649	0.885436
MED6	1.025971	0.721017	1.459906	0.88671
DGKD	0.984552	0.79152	1.224659	0.888796
NFKB1	1.018508	0.786647	1.318709	0.889334
SUMO2	1.023575	0.735388	1.424696	0.890148
SLC27A5	0.984016	0.781703	1.238692	0.890869
MED25	0.978495	0.711774	1.345162	0.893493
FABP3	1.011209	0.858236	1.191449	0.894037
HELZ2	1.011935	0.848678	1.206596	0.894853
PIK3R3	0.984597	0.781994	1.239692	0.89494
PCTP	1.016117	0.79804	1.293785	0.896795
DGAT2L6	0.997119	0.954025	1.042161	0.898172
CYP7A1	0.997056	0.952308	1.043907	0.899868
CRAT	0.991656	0.869746	1.130655	0.900372
PCYT1B	1.006961	0.902244	1.123832	0.901459
GM2A	0.982828	0.745872	1.295062	0.90206
ENPP7	1.002835	0.957624	1.05018	0.904276
NFYC	0.980008	0.699906	1.372207	0.906398
SOAT1	0.988121	0.809407	1.206294	0.906543
SGPL1	1.016188	0.777021	1.32897	0.906635
ACACB	0.990932	0.850315	1.154804	0.907129
KDSR	1.016518	0.763272	1.353788	0.91077
UGT2B15	1.003899	0.937542	1.074952	0.911203
PRKAB1	1.018197	0.740389	1.400244	0.911669
SLC27A4	0.985439	0.760164	1.277474	0.911805
DGKI	1.008437	0.86413	1.176844	0.91508
DGKG	1.008973	0.856102	1.189142	0.915134
SMPD2	0.984869	0.74261	1.306158	0.915706
PNLIPRP1	1.002061	0.964233	1.041373	0.916477
CYP2R1	0.98392	0.725549	1.334297	0.916927
CYP51A1	1.009277	0.847764	1.20156	0.917342
VAC14	0.98373	0.720424	1.343272	0.917798
DEGS1	1.010546	0.827825	1.233597	0.917891
CERS5	1.019379	0.707389	1.46897	0.917993
MFSD2A	1.006889	0.883038	1.148111	0.918343
CYP2C8	0.995192	0.907478	1.091386	0.918463
MED8	1.018317	0.715329	1.449638	0.919763
PLD6	1.009999	0.829894	1.229189	0.920915
OSBPL5	1.014206	0.763431	1.347355	0.92246
PIK3R5	0.991348	0.830891	1.182791	0.923154
ACOT7	1.011091	0.806793	1.267121	0.9237
ST3GAL3	0.985138	0.715285	1.356796	0.926949
PI4KB	1.015566	0.72608	1.420471	0.928108
ALDH3A2	0.991305	0.817535	1.20201	0.929231
RDH11	1.013339	0.756188	1.357939	0.929299
HSD17B11	1.009472	0.819253	1.243857	0.929483
TNFAIP8L3	1.00709	0.85955	1.179955	0.930343
CYP11B2	0.997442	0.941131	1.057123	0.931168
MBOAT1	1.006606	0.861285	1.176447	0.934031
B4GAT1	1.01155	0.767935	1.332448	0.934893
SGMS1	1.009769	0.798864	1.276355	0.93518
GPAT3	0.993962	0.85617	1.153931	0.936604
UGT2B17	0.99788	0.947025	1.051466	0.936623
EPHX1	0.991866	0.80213	1.226483	0.939901

PRKACG	0.998326	0.955072	1.043538	0.940888
SRD5A1	1.007491	0.827001	1.227373	0.940932
ELOVL3	1.004002	0.902824	1.116519	0.941255
GK	1.008131	0.810294	1.254271	0.942078
PIK3CG	0.995292	0.875134	1.131948	0.94269
MED22	1.010173	0.760372	1.342041	0.944325
ACADSB	0.994506	0.848318	1.165885	0.945849
CYP1A1	1.001628	0.951041	1.054905	0.950959
PLAAT1	1.002789	0.91706	1.096533	0.951288
FADS2	0.99684	0.900348	1.103673	0.951408
PLD1	0.994469	0.828808	1.193242	0.95243
PON2	0.992562	0.759675	1.296843	0.95636
MED27	0.991148	0.707603	1.388312	0.958755
B3GNT4	1.004675	0.836852	1.206153	0.96011
MTMR14	1.008381	0.725844	1.400898	0.960315
MDH2	0.993828	0.738479	1.33747	0.967406
PRKD3	1.003602	0.842511	1.195494	0.967872
B4GALNT1	1.002832	0.87081	1.154868	0.968682
MED30	0.995434	0.777663	1.274188	0.971018
ACSM6	1.00086	0.952015	1.052211	0.973151
INPPL1	1.004316	0.749133	1.346424	0.977028
PLPP3	0.997126	0.811202	1.225665	0.978195
BPHL	1.003558	0.740146	1.360715	0.981759
OSTC	1.003741	0.724463	1.39068	0.982092
UGT1A7	0.999236	0.934598	1.068344	0.982129
CSNK2A2	1.003064	0.74342	1.35339	0.98403
MED10	0.996736	0.715274	1.388953	0.984593
PLA2R1	1.001739	0.805335	1.246042	0.987549
ARSG	0.999036	0.837439	1.191816	0.991453
MAPK8	0.998774	0.758065	1.315916	0.993045
FA2H	1.000539	0.870938	1.149425	0.993929
SPHK2	1.000497	0.759588	1.317813	0.997178
SMPD3	0.99984	0.873607	1.144313	0.998146
ACSS1	1.000084	0.845417	1.183047	0.99922
