# Supplementary material for: Exploration of prognosis and immunometabolism landscapes in ER+ breast cancer based on a novel lipid metabolism-related signature
Source: Front Immunol. 2023 Jul 4;14:1199465. doi: 10.3389/fimmu.2023.1199465 (PMC10352658; doi:10.3389/fimmu.2023.1199465)
Supplement: Supplementary file 6 [file Table_5.pdf]

Genes	p.value	HR	Low 95%CI	High 95%CI
FIG4	1.74E-05	2.545856	1.6621372	3.8994256
OCRL	0.0002	2.203484	1.4530075	3.3415808
INPP5F	0.001632	1.955129	1.2882654	2.96719
CPT1A	0.001921	1.899463	1.2664496	2.8488769
FAM120B	0.002169	1.887665	1.2575852	2.8334296
MMUT	0.002498	1.886319	1.2501324	2.8462575
ALOX15	0.003065	1.857917	1.2329879	2.7995859
PRKAB2	0.002933	1.847851	1.2330026	2.7692999
AHRR	0.003792	1.82959	1.2154475	2.7540474
OSBPL10	0.003682	1.815757	1.2140242	2.7157409
CHUK	0.006971	1.761926	1.1676316	2.6587009
ACBD5	0.012212	1.678285	1.1194191	2.5161615
CCNC	0.014279	1.668597	1.1078611	2.5131449
PTGES3	0.015405	1.647285	1.1000266	2.4668026
MBTPS2	0.024432	1.58981	1.0616353	2.380758
CLOCK	0.025952	1.586016	1.0568485	2.3801384
SYNJ2	0.024885	1.582207	1.0596395	2.3624817
HIBCH	0.027527	1.577847	1.0517875	2.3670177
PLEKHA3	0.02975	1.575347	1.0456435	2.3733882
HSP90AA1	0.026945	1.563051	1.0522662	2.3217781
APOA5	0.03116	1.547986	1.0403434	2.3033361
RAB5A	0.036698	1.538705	1.0269985	2.305371
PLA2G12A	0.035672	1.534574	1.0290949	2.2883379
STARD3NL	0.036746	1.530388	1.026549	2.2815148
MED23	0.045055	1.504792	1.009048	2.2440934
CYP27A1	0.045307	0.666178	0.4475755	0.9915478
FUT6	0.041332	0.661426	0.4446531	0.9838779
HPGD	0.038761	0.658209	0.4427003	0.9786285
PLPPR1	0.037525	0.655768	0.4406315	0.9759429
CYP2D6	0.033301	0.645756	0.4316881	0.9659764
PIK3CD	0.024264	0.629843	0.4212804	0.9416583
PLEKHA4	0.02249	0.628519	0.4217909	0.936569
ADH6	0.020066	0.624196	0.4195533	0.9286574
PRKCQ	0.018741	0.614003	0.4088344	0.9221324
PLA2G2D	0.017308	0.612953	0.4096168	0.9172257
PLD4	0.018629	0.612225	0.4068237	0.9213327
SLCO1A2	0.012219	0.599181	0.4013901	0.8944352
APOA1	0.013238	0.598297	0.3984927	0.8982818
ALDH3A1	0.01098	0.597432	0.40169	0.8885577
CBR3	0.01139	0.595413	0.3984804	0.8896729
S1PR5	0.010493	0.589148	0.3928685	0.8834889
CYP24A1	0.008874	0.588216	0.3953002	0.8752788
NFKBIE	0.005109	0.562947	0.3765338	0.8416501
NFKBIA	0.004202	0.557633	0.3738317	0.8318037
CHKB	0.005352	0.557367	0.3693946	0.8409906
CERS3	0.002471	0.539415	0.3616911	0.8044663
SLC27A6	0.002193	0.533298	0.3566583	0.7974211
CA6	0.000792	0.496717	0.3300748	0.7474897
S1PR4	0.000849	0.486403	0.3185064	0.7428053
FABP7	0.000553	0.484222	0.3208545	0.7307694
