# Supplementary material for: Exploration of prognosis and immunometabolism landscapes in ER+ breast cancer based on a novel lipid metabolism-related signature
Source: Front Immunol. 2023 Jul 4;14:1199465. doi: 10.3389/fimmu.2023.1199465 (PMC10352658; doi:10.3389/fimmu.2023.1199465)
Supplement: Supplementary file 7 [file Table_6.pdf]

Gene-CpG	HR	CI	p
HIBCH-TSS200-Island-cg01027365	2.026	1.229-3.341	0.0031
HIBCH-Body-N_Shore-cg16814399	0.598	0.397-0.901	0.017
OSBPL10-Body-Open_Sea-cg02057211	0.57	0.357-0.908	0.013
OSBPL10-Body-Open_Sea-cg02099390	0.545	0.33-0.899	0.012
OSBPL10-Body-Open_Sea-cg04370634	0.544	0.369-0.802	0.0023
OSBPL10-Body-Open_Sea-cg10789050	0.456	0.307-0.676	0.00014
OSBPL10-Body-Open_Sea-cg22259536	0.62	0.376-1.021	0.049
OSBPL10-Body-Open_Sea-cg22539670	0.512	0.325-0.805	0.0023
OSBPL10-Body-Open_Sea-cg24264962	0.501	0.332-0.755	0.00068
OSBPL10-Body-Open_Sea-cg24391989	0.467	0.291-0.748	0.00072
OSBPL10-Body-Open_Sea-cg27298830	0.598	0.404-0.884	0.011
FIG4-Body-S_Shelf-cg03492495	0.619	0.421-0.912	0.016
FIG4-Body-Open_Sea-cg04940695	0.587	0.399-0.865	0.0075
FIG4-Body-Open_Sea-cg16019558	0.545	0.358-0.931	0.0068
OCRL-TSS200-Island-cg05032353	0.611	0.406-0.919	0.022
OCRL-TSS200-Island-cg27633753	0.621	0.42-0.918	0.019
OCRL-5'UTR;1stExon-Island-cg05147108	0.494	0.335-0.728	0.00038
OCRL-5'UTR;1stExon-Island-cg16716035	0.436	0.294-0.646	0.000035
OCRL-Body-Island-cg13532816	0.641	0.405-1.013	0.048
OCRL-3'UTR-Open_Sea-cg06055803	1.666	1.113-2.494	0.012
CPT1A-5'UTR-N_Shore-cg00574958	0.417	0.282-0.616	0.000011
CPT1A-5'UTR-N_Shore-cg17058475	0.436	0.294-0.648	0.000033
CPT1A-5'UTR-N_Shore-cg19081843	1.752	1.166-2.633	0.0058
CPT1A-5'UTR-N_Shore-cg23756264	0.529	0.317-0.883	0.0094
CPT1A-TSS200-N_Shore-cg10132543	1.733	1.118-2.685	0.011
CPT1A-Body-N_Shore-cg10703936	0.652	0.422-1.007	0.047
CPT1A-Body-N_Shore-cg23406642	0.66	0.445-0.979	0.042
CPT1A-TSS1500-N_Shore-cg13786863	0.608	0.361-1.023	0.048
CPT1A-TSS1500-N_Shore-cg15616358	0.604	0.385-0.948	0.023
CPT1A-TSS1500-N_Shore-cg24366211	0.552	0.328-0.93	0.017
CPT1A-5'UTR-Island-cg01082498	0.613	0.385-0.975	0.031
CPT1A-5'UTR-Island-cg09806934	0.665	0.45-0.983	0.039
CPT1A-5'UTR-Island-cg26989316	0.492	0.333-0.725	0.00035
CPT1A-Body-Island-cg10553894	0.483	0.279-0.837	0.0049
CPT1A-Body-Open_Sea-cg01926073	0.563	0.381-0.832	0.0038
CPT1A-Body-Open_Sea-cg03855388	0.434	0.293-0.642	0.000026
CPT1A-Body-Open_Sea-cg04732324	0.49	0.332-0.724	0.00034
CPT1A-Body-Open_Sea-cg05710301	0.529	0.356-0.787	0.0022
CPT1A-Body-Open_Sea-cg06042565	0.602	0.405-0.896	0.011
CPT1A-Body-Open_Sea-cg13491471	0.562	0.377-0.838	0.0041
CPT1A-Body-Open_Sea-cg18262591	0.556	0.372-0.833	0.0058
CPT1A-Body-Open_Sea-cg20629021	0.526	0.355-0.779	0.0012
CPT1A-Body-Open_Sea-cg20809737	0.522	0.348-0.782	0.0024
CPT1A-Body-Open_Sea-cg26906629	0.51	0.346-0.754	0.0077
CPT1A-5'UTR-Open_Sea-cg20285002	0.459	0.311-0.678	0.000098
CPT1A-3'UTR;Body-S_Shelf-cg02146941	0.517	0.307-0.871	0.0078
CPT1A-Body-N_Shelf-cg21868480	0.65	0.44-0.961	0.03
CPT1A-5'UTR-N_Shelf-cg22911054	0.621	0.418-0.922	0.02
INPP5F-Body;TSS1500-Island-cg02722214	0.61	0.405-0.919	0.021
INPP5F-Body;TSS1500-Island-cg08338216	0.535	0.355-0.806	0.0039
INPP5F-Body;TSS1500-Island-cg11613559	0.641	0.422-0.973	0.042
INPP5F-Body;TSS1500-Island-cg20365618	0.654	0.441-0.972	0.039
INPP5F-Body;TSS1500-S_Shore-cg0285755	0.568	0.383-0.841	0.0055
INPP5F-Body-S_Shore-cg15922976	0.619	0.419-0.912	0.015
INPP5F-Body-S_Shore-cg19978242	0.57	0.387-0.841	0.0049
INPP5F-Body;TSS200-S_Shore-cg2504578	0.597	0.4-0.89	0.013
INPP5F-3'UTR;Body-Open_Sea-cg0390467	0.59	0.358-0.973	0.03

INPP5F-Body-Open_Sea-cg07679322	0.54	0.362-0.804	0.0032
INPP5F-Body-Open_Sea-cg10053779	0.605	0.368-0.997	0.038
INPP5F-Body-Open_Sea-cg11640046	1.664	1.096-2.528	0.014
INPP5F-TSS1500-N_Shore-cg10149021	0.507	0.34-0.755	0.0012
INPP5F-TSS1500-N_Shore-cg18425877	0.545	0.369-0.805	0.0027
INPP5F-TSS1500-N_Shore-cg27613076	0.535	0.36-0.795	0.0019
PTGES3-TSS1500-Island-cg20253639	1.787	1.016-3.144	0.031
PTGES3-3'UTR-Open_Sea-cg13853217	0.558	0.352-0.884	0.0093
HSP90AA1-Body-Open_Sea-cg05412906	0.487	0.301-0.788	0.0018
HSP90AA1-Body-Open_Sea-cg13263472	0.651	0.434-0.975	0.042
HSP90AA1-5'UTR;Body-Island-cg06798479	2.446	1.391-4.301	0.00057
HSP90AA1-5'UTR;Body-Island-cg21735344	0.588	0.376-0.917	0.025
HSP90AA1-TSS1500;Body-Island-cg2330488	1.705	1.044-2.787	0.025
HSP90AA1-Body-N-Shore-cg11789371	0.653	0.443-0.962	0.031
HSP90AA1-TSS1500;Body-S-Shore-cg1379	1.635	1.005-2.658	0.038
ALOX15-Body-Island-cg06222638	0.648	0.426-0.986	0.049
ALOX15-1stExon-Island-cg09872233	1.74	1.136-2.664	0.0084
ALOX15-Body-N_Shore-cg11609940	0.544	0.368-0.802	0.0025
