# Supplementary material for: Exploration of prognosis and immunometabolism landscapes in ER+ breast cancer based on a novel lipid metabolism-related signature
Source: Front Immunol. 2023 Jul 4;14:1199465. doi: 10.3389/fimmu.2023.1199465 (PMC10352658; doi:10.3389/fimmu.2023.1199465)
Supplement: Supplementary file 8 [file Table_7.pdf]

gene	lowMean	highMean	logFC	pValue	fdr
LORICRIN	248.7232	1.845387	-7.074474	8.21E-09	2.34E-08
C4BPA	233.4115	27.19701	-3.101356	0.000354	0.000626
SYT14	26.29177	69.62344	1.404962	0.02053	0.028252
LEMD1	21.56608	8.029925	-1.425306	6.08E-05	0.000118
BLACAT1	31.6783	13.85037	-1.19357	5.32E-06	1.16E-05
BGLAP	72.28928	23.4015	-1.627181	3.94E-14	1.71E-13
PADI3	63.49377	128.6559	1.01883	0.007226	0.010682
RNF186	13.88279	6.887781	-1.011187	0.008572	0.012522
CD52	2365.471	978.0998	-1.274074	0.010807	0.015544
H2AC20	92.37905	36.68329	-1.332443	7.75E-08	2.02E-07
TRIM67	59.03741	126.1421	1.095349	5.65E-05	0.00011
ETV3L	8.428928	3.793017	-1.152003	1.82E-06	4.17E-06
IL19	45.97756	96.60349	1.071146	0.000631	0.001082
VWA5B1	6.069825	15.71072	1.372023	0.002635	0.004149
ALKAL2	47.87781	12.81796	-1.901191	0.018585	0.025745
BCL11A	329.808	161.3641	-1.031307	5.04E-05	9.89E-05
SLC9A2	215.3616	607.7531	1.496724	2.45E-10	7.88E-10
TSPYL6	2.588529	7.910224	1.611586	0.003352	0.005195
SLC5A7	14.97506	88.09476	2.556494	0.035054	0.046307
EN1	305.4214	148.8653	-1.036794	0.000991	0.001654
SLC4A10	83.6808	274.3541	1.713071	3.62E-05	7.22E-05
WNT6	48.86534	22.00499	-1.150981	6.85E-12	2.48E-11
CNGA3	48.96758	148.4838	1.600407	0.000361	0.000638
C2orf50	77.70075	219.0698	1.49539	5.32E-09	1.54E-08
KLHL41	45.08479	107.4389	1.252804	0.00546	0.00822
USP37	706.3017	1435.706	1.023404	1.78E-44	6.61E-41
POMC	43.49377	19.37905	-1.166311	7.95E-13	3.11E-12
RTP5	5.588529	2.658354	-1.071935	2.63E-07	6.53E-07
ASPRV1	155.7057	77.68579	-1.0031	1.60E-06	3.68E-06
SCN1A	34.68329	80.85037	1.221014	0.036789	0.048431
NEB	145.0798	442.9975	1.610452	0.000177	0.000326
KCNH7	3.660848	11.43142	1.642755	0.000102	0.000193
PCARE	6.882793	2.855362	-1.269321	0.003639	0.005613
AOX1	577.1322	1194.09	1.048938	0.000554	0.000957
CADPS	51.58105	238.0998	2.206653	0.029053	0.03893
CLDN18	11.68828	29.14963	1.318415	0.013962	0.019717
CLSTN2	10824.23	23737.7	1.132915	9.58E-12	3.43E-11
LMOD3	15.28678	39.61845	1.373888	0.011044	0.015871
STXBP5L	12.91022	46.71072	1.85524	5.57E-06	1.22E-05
PEX5L	186.5985	705.8579	1.91944	1.68E-11	5.93E-11
FGF12	229.4464	551.2219	1.264476	4.65E-05	9.16E-05
FRG2C	3.598504	8.865337	1.300778	0.001044	0.001737
EPHA6	23.13965	52.83791	1.191206	1.02E-06	2.40E-06
ROPN1	68.2818	23.06983	-1.565494	6.86E-07	1.64E-06
TACR3	9.13217	19.08728	1.063582	1.97E-06	4.48E-06
UGT2B4	353.7232	1148.297	1.698803	0.000711	0.001211
AFP	83.87032	351.0773	2.065556	0.018641	0.025811
NDST4	70.3616	154.6608	1.136248	3.03E-07	7.47E-07
FGFBP1	108.2095	27.29676	-1.987025	0.000507	0.00088
FGFBP2	63.19202	21.6384	-1.546148	0.000443	0.000776
WDR17	138.0449	282.0948	1.031042	0.000174	0.00032
FRAS1	369.5062	761.3491	1.042959	5.03E-08	1.33E-07
ART3	89.37406	42.14464	-1.084507	0.013115	0.018605
CXCL1	105.0898	41.79551	-1.330202	0.011874	0.016978
CXCL5	59.99501	13.95511	-2.104049	0.036701	0.048324
FDCSP	2206.559	195.3267	-3.497837	5.38E-06	1.18E-05
CDH18	28.13217	75.42893	1.422897	0.000178	0.000328

GDNF	31.10973	228.8703	2.879093	0.02321	0.031629
PCDHAC2	33.49127	77.399	1.20853	7.26E-12	2.63E-11
GMCL2	0.822943	1.798005	1.127533	1.83E-06	4.19E-06
POU4F3	5.159601	34.63342	2.746833	0.033435	0.044322
FABP7	585.2095	164.6783	-1.829303	6.92E-05	0.000133
GJB7	9.204489	20.91022	1.183799	2.13E-06	4.83E-06
IYD	122.8329	285.5935	1.217266	2.52E-10	8.12E-10
C6orf15	373.2095	15.45636	-4.593713	0.001796	0.002896
PSORS1C2	92.12469	6.094763	-3.917946	0.032782	0.043538
LTB	685.01	266.9052	-1.359797	4.45E-11	1.52E-10
CRISP3	1214.07	4299.663	1.824372	0.007012	0.010391
KIAA0319	70.16209	140.9551	1.006472	1.21E-10	3.97E-10
APOBEC2	6.254364	14.85786	1.248291	1.49E-08	4.15E-08
CRISP1	3.077307	17.74564	2.527724	0.000501	0.00087
COL9A1	50.82544	8.139651	-2.642512	0.019971	0.027547
HS3ST5	17.56608	55.40648	1.657262	0.000616	0.001057
MEI4	22.90524	47.00249	1.03706	0.001183	0.001956
SCIN	864.6633	1821.454	1.07488	0.035106	0.046359
ZNF804B	10.6808	23.32668	1.126962	1.22E-05	2.56E-05
DLX5	109.4414	51.6384	-1.083642	0.00011	0.000208
CLEC2L	14.89027	6.870324	-1.11592	0.000696	0.001186
NPC1L1	85.42145	193.5387	1.179951	1.44E-09	4.36E-09
VSTM2A	1379.274	2861.905	1.053066	1.24E-05	2.61E-05
ASB15	5.518703	11.95012	1.114624	0.000658	0.001127
POF1B	417.4214	841.1721	1.010896	3.49E-07	8.56E-07
GABRA3	33.89027	71.86783	1.084475	0.019676	0.027167
NEXMIF	189.8803	396.4165	1.061927	3.80E-20	2.82E-19
PCDH19	517.0399	1134.1	1.1332	4.98E-09	1.44E-08
GDPD2	33.37656	97.21197	1.542299	1.98E-11	6.96E-11
RTL9	28.15461	230.4913	3.033269	2.87E-06	6.43E-06
GRIA3	108.0574	244.0249	1.175231	4.15E-05	8.23E-05
PNMA3	46.82793	21.98753	-1.090684	4.18E-08	1.12E-07
GABRQ	103.4638	464.3317	2.166029	0.008583	0.012537
CNGA2	11.70075	39.25187	1.746161	2.06E-05	4.23E-05
PEBP4	254.98	104.1546	-1.291658	1.18E-05	2.48E-05
DEFB1	165.0274	69.48878	-1.247854	4.22E-05	8.36E-05
RAB11FIP1	6913.923	13875.68	1.004982	7.10E-22	6.34E-21
CRISPLD1	2098.766	5321.007	1.342158	0.00857	0.01252
PXDNL	398.8603	1105.903	1.471269	6.80E-10	2.12E-09
TRPA1	716.7805	1999.85	1.480289	0.000433	0.000758
WWP1	10119.67	20882.82	1.045154	2.12E-38	5.50E-36
RIMS2	168.813	412.2269	1.288013	0.000273	0.00049
LY6D	603.0274	79.16958	-2.929206	3.62E-06	8.03E-06
FAM135B	133.2195	294.8728	1.146288	1.25E-06	2.91E-06
CSMD3	82.11471	244.9027	1.576496	6.99E-07	1.67E-06
CHRNA6	58.15711	129.5411	1.155383	0.001285	0.002113
TTPA	35.23192	71.01496	1.01124	1.22E-10	4.00E-10
PI15	2853.608	7571.399	1.407772	0.008725	0.012728
SCX	88.30673	42.7207	-1.047588	3.57E-11	1.23E-10
KCNK9	10.05486	23.33167	1.214396	0.010213	0.014747
CNGB3	6.561097	15.44389	1.235027	8.01E-21	6.37E-20
CCNE2	336.7805	726.8504	1.10985	1.19E-30	3.87E-29
SLC30A8	2779.481	7554.224	1.442468	2.01E-09	5.99E-09
GATA4	67.65835	136.399	1.011493	0.007112	0.010532
HRCT1	129.4439	52.99501	-1.288398	3.96E-10	1.26E-09
ORM2	112.9776	51.94763	-1.120906	0.037217	0.048946
TAF1L	2.55611	6.678304	1.385532	0.002713	0.004261
OBP2B	689.8429	337.7082	-1.03049	1.01E-05	2.14E-05

ANKRD18B	7.513716	23.62095	1.652468	8.42E-06	1.81E-05
SCGB1D2	34058.64	11452.61	-1.572345	0.002064	0.003301
EEF1G	33.93017	16.82045	-1.012353	1.11E-23	1.21E-22
SCGB2A1	3553.628	614.6384	-2.531483	0.002277	0.003615
RAB39A	26.74564	54.35162	1.02302	1.09E-11	3.88E-11
KCNC1	95.95012	194.1222	1.016608	0.003949	0.006058
TMPRSS4	347.1471	733.5686	1.079385	0.000227	0.000411
SAA2-SAA4	120.2718	59.28429	-1.020577	2.35E-05	4.80E-05
FAM181B	135.2344	61.08728	-1.146518	3.92E-07	9.57E-07
NCR3LG1	150.8828	307.7357	1.028263	1.45E-23	1.57E-22
CHRM4	19.42394	40.23441	1.050594	0.000149	0.000277
SLC22A24	3.441397	8.977556	1.383329	5.33E-08	1.41E-07
KCNK7	34.30424	12.7581	-1.426973	8.69E-07	2.06E-06
CHRDL2	368.1421	51.18204	-2.846553	0.003991	0.006119
MYBPC3	14.61596	5.274314	-1.470489	0.000171	0.000315
IZUMO1R	2.995012	1.309227	-1.193847	1.26E-08	3.53E-08
SLC22A25	2.950125	9.19202	1.639606	1.46E-05	3.04E-05
SYT8	329.9476	118.4913	-1.477456	3.64E-10	1.16E-09
ACBD7	38.43641	82.73815	1.106079	4.46E-20	3.29E-19
SLIT1	194.02	807.1671	2.056662	0.00015	0.000278
LGI1	23.85786	67.81546	1.50715	0.013673	0.019333
ACSM6	4.633416	10.49127	1.179041	0.000664	0.001137
COMMD3-BMI1	4.21197	9.53616	1.178913	3.91E-08	1.05E-07
FAM234B	5873.337	12159.81	1.049868	6.52E-23	6.42E-22
SYT10	3.950125	22.24439	2.493471	4.06E-08	1.08E-07
KRT6B	2308.092	600.2419	-1.943085	0.001569	0.002551
AQP5	610.3392	224.2793	-1.444315	0.000151	0.00028
KRT86	347.0224	148.6908	-1.222714	0.000464	0.00081
KRT5	11046.99	5364.985	-1.042007	3.00E-05	6.03E-05
E2F7	211.8279	437.788	1.04734	6.66E-24	7.46E-23
ASCL1	314.0723	787.7855	1.326706	1.09E-05	2.32E-05
RPH3A	8.957606	21.09975	1.236041	0.034063	0.045066
OTOGL	38.52369	91.52868	1.248478	1.72E-10	5.60E-10
KRT79	77.10973	12.33167	-2.644545	0.005153	0.00779
KRT81	2489.953	732.1372	-1.765932	6.21E-07	1.49E-06
OVCH1	5.710723	17.3192	1.600627	0.018354	0.025451
GPR12	60.24688	14.48878	-2.055951	0.021528	0.029505
FREM2	272.217	754.6484	1.471048	1.37E-05	2.87E-05
OLFM4	4021.087	1667.055	-1.270284	0.004925	0.007464
HS6ST3	599.4239	1376.002	1.198834	1.10E-09	3.35E-09
LRRC9	5.416459	11.14464	1.040928	4.57E-06	1.01E-05
TCL1B	7.561097	21.27681	1.492614	0.000181	0.000333
CMTM5	15.37157	5.321696	-1.530307	0.000143	0.000267
TCL1A	118.4813	50.59352	-1.227635	0.02771	0.037251
SMOC1	458.4863	163.3117	-1.48925	0.002246	0.003569
ZDHHC22	20.91022	78.72319	1.91258	0.0024	0.003797
EML5	23.07232	58.74065	1.348196	1.15E-16	6.19E-16
TUNAR	45.40648	20.73067	-1.131131	0.008873	0.012927
OCA2	20.76808	7.179551	-1.532402	0.0053	0.007998
HCN4	12.63591	34.15711	1.434656	0.005681	0.008535
WDR72	218.7207	557.9776	1.351117	0.01356	0.019191
RHCG	195.5411	35.59102	-2.457887	0.002291	0.003636
SLC28A2	15.90025	37.72319	1.246402	0.013591	0.019228
TGM5	90.6783	33.51122	-1.436113	0.000115	0.000216
SHISA9	377.3267	838.0623	1.151243	6.00E-15	2.79E-14
MYLK3	20.05237	42.02494	1.067473	1.46E-09	4.41E-09
SYCE1L	68.50873	30.41895	-1.171317	3.51E-16	1.82E-15
CD19	131.419	58.01746	-1.179614	0.000691	0.001179

GDPD3	365.2469	850.2968	1.219094	0.017234	0.02401
GPR139	17.44888	47.35661	1.440432	1.84E-07	4.64E-07
PRSS33	59.50374	3.034913	-4.293253	0.009178	0.013336
SOX8	194.98	57.59601	-1.759286	1.54E-08	4.27E-08
SLC6A4	798.4464	1668.559	1.063335	0.003145	0.004891
PNMT	1194.012	204.2319	-2.547538	0.001259	0.002071
LPO	39.41895	11.28429	-1.804574	0.035314	0.046622
IGF2BP1	16.95761	55.86534	1.720021	0.003835	0.005899
TNFRSF13B	45.02244	21.28678	-1.080686	6.80E-07	1.63E-06
B4GALNT2	132.788	273.5885	1.042883	0.002289	0.003633
TEX19	13.64589	27.56858	1.014559	5.56E-09	1.60E-08
PRR11	736.0125	1686.451	1.196189	9.92E-13	3.86E-12
ALOX15	66.70823	328.2469	2.298845	1.46E-25	2.04E-24
KRT24	21.70823	51.22943	1.238731	0.009743	0.014104
CD79B	396.1247	197.0623	-1.007302	3.53E-08	9.47E-08
LHX1	44.86534	115.9177	1.369428	0.000272	0.000489
KRT14	8912.516	3820.197	-1.222186	5.45E-08	1.44E-07
KRT16	1321.045	355.3242	-1.894472	3.65E-07	8.94E-07
KLHL11	189.2843	385.0823	1.024612	7.56E-42	6.37E-39
CELF4	59.27431	141.783	1.258206	0.006233	0.009295
ONECUT2	114.4165	290.9576	1.346514	0.000156	0.00029
DIPK1C	45.66085	19.80798	-1.204876	5.58E-05	0.000109
LRRN4	15.20948	35.32668	1.215788	0.029434	0.039402
PI3	216.9127	77.74065	-1.480374	0.003415	0.005287
GDF5	45.64838	16.27681	-1.487746	2.88E-05	5.82E-05
RIMS4	1654.065	3364.653	1.024442	1.69E-07	4.27E-07
MMP9	8820.072	4377.773	-1.010593	0.015453	0.021675
COL9A3	408.2993	172.2793	-1.244878	1.95E-09	5.84E-09
C20orf141	2.805486	1.27182	-1.141356	0.004984	0.007549
ZNF729	9.416459	31.62594	1.747852	3.72E-06	8.25E-06
CYP2A6	2193.988	6289.09	1.519296	0.000189	0.000346
CYP2B6	15.41646	36.01496	1.224125	1.36E-05	2.86E-05
KRTDAP	411.187	14.41397	-4.834256	0.007148	0.010582
MIA	152.7007	44.70574	-1.772175	2.83E-09	8.35E-09
SBSN	512.0125	30.94514	-4.048394	0.007904	0.011611
ATP1A3	177.6459	404.616	1.187549	0.002365	0.003747
FCER2	74	22.89776	-1.692319	7.63E-05	0.000146
GZMM	131.9252	61.38903	-1.103667	3.75E-10	1.19E-09
LGALS7B	256.9825	15.1596	-4.083367	0.014513	0.020431
GFY	4.541147	16.24439	1.838813	4.35E-05	8.60E-05
LGALS7	19.04239	8.74813	-1.122168	0.000175	0.000323
EFNA2	4.082294	8.261845	1.017084	0.004837	0.007337
PEG3	1306.875	3498.601	1.420657	1.80E-08	4.97E-08
ZIM2	6.456359	18.02993	1.481601	2.81E-09	8.29E-09
KLK6	484.202	156.1995	-1.632219	0.002106	0.003363
KLK7	381.8105	166.7681	-1.195014	0.00516	0.007799
FAM187B	7.912718	0.82793	-3.256592	0.000236	0.000426
IGLL1	8.40399	2.743142	-1.615245	0.00294	0.004593
ELFN2	68.8005	146.4688	1.090103	0.00816	0.011963
USP41	11.05985	27.96259	1.338166	8.80E-11	2.93E-10
MLC1	126.1372	44.4414	-1.505017	0.010652	0.015336
DSCAM	15.21197	33.25935	1.128553	0.036249	0.047758
