# Supplementary material for: Exploration of prognosis and immunometabolism landscapes in ER+ breast cancer based on a novel lipid metabolism-related signature
Source: Front Immunol. 2023 Jul 4;14:1199465. doi: 10.3389/fimmu.2023.1199465 (PMC10352658; doi:10.3389/fimmu.2023.1199465)
Supplement: Supplementary file 9 [file Table_8.pdf]

id	GeneClass
STAT1	STAT1
GBP1	STAT1
TAP1	STAT1
IRF1	STAT1
CXCL9	STAT1
PSMB9	STAT1
CXCL10	STAT1
CXCL11	STAT1
IFIH1	STAT1
HLA-F	MHC-I
HLA-B	MHC-I
HLA-G	MHC-I
HLA-A	MHC-I
HLA-C	MHC-I
HLA-DPB1	MCH-II
CTSS	MCH-II
HLA-DMB	MCH-II
HLA-DRB1	MCH-II
HLA-DRA	MCH-II
CD74	MCH-II
HLA-DPA1	MCH-II
HLA-DQA1	MCH-II
HLA-DMA	MCH-II
CCL5	LCK
IL2RG	LCK
CD48	LCK
SELL	LCK
LCK	LCK
GZMA	LCK
IL7R	LCK
KLRK1	LCK
CD2	LCK
STAT4	LCK
SLAMF1	LCK
CCR7	LCK
GZMK	LCK
CCR2	LCK
SH2D1A	LCK
ITK	LCK
CD3D	LCK
GIMAP5	LCK
PLAC8	LCK
GIMAP4	LCK
PRG1	LCK
HCLS1	LCK
INPP5D	LCK
CD53	LCK
SLA	LCK
PIK3CD	LCK
IRF8	LCK
FGL2	LCK
IL10RA	LCK
CSF2RB	LCK
LCP2	LCK
CORO1A	LCK
SELPLG	LCK
EVI2B	LCK

PTPRC	LCK
RAC2	LCK
LPXN	LCK
ARHGAP15	LCK
SAMSN1	LCK
MX1	Interferon
IFI27	Interferon
OAS1	Interferon
IFIT1	Interferon
IFI44L	Interferon
IFIT3	Interferon
OAS2	Interferon
RSAD2	Interferon
OAS3	Interferon
IFI30	HCK
LAPTM5	HCK
ITGB2	HCK
C1QB	HCK
CD163	HCK
TYROBP	HCK
FCER1G	HCK
CCR1	HCK
TFEC	HCK
NCF2	HCK
LAIR1	HCK
CD86	HCK
C1QA	HCK
MS4A4A	HCK
MNDA	HCK
AIF1	HCK
LST1	HCK
DOCK2	HCK
RNASE6	HCK
MS4A6A	HCK
